# Altered Moesin and Actin Cytoskeleton Protein Rearrangements Affect Transendothelial Permeability in Human Endothelial Cells upon Dengue Virus Infection and TNF-α Treatment

**DOI:** 10.3390/v13102042

**Published:** 2021-10-11

**Authors:** Aroonroong Suttitheptumrong, Thanaporn Mahutchariyakul, Nantapon Rawarak, Onrapak Reamtong, Kobporn Boonnak, Sa-nga Pattanakitsakul

**Affiliations:** 1 Division of Molecular Medicine, Research Department, Faculty of Medicine Siriraj Hospital, Mahidol University, Bangkok 10700, Thailand; aroonroong.sut@mahidol.ac.th (A.S.); ferin.mii@gmail.com (T.M.); n_1103@hotmail.com (N.R.); 2 Graduate Program in Immunology, Department of Immunology, Faculty of Medicine Siriraj Hospital, Mahidol University, Bangkok 10700, Thailand; 3 Department of Molecular Tropical Medicine and Genetics, Faculty of Tropical Medicine, Mahidol University, Bangkok 10400, Thailand; onrapak.rea@mahidol.ac.th; 4 Department of Microbiology and Immunology, Faculty of Tropical Medicine, Mahidol University, Bangkok 10400, Thailand; kboonnak@gmail.com

**Keywords:** cytoskeleton protein, dengue virus infection, TNF-alpha, transendothelial permeability

## Abstract

It has been hypothesized that the host, viral factors, and secreted cytokines (especially TNF-α) play roles in the pathogenesis of secondary dengue infections. Mass spectrometry-based proteomic screening of cytoskeleton fractions isolated from human endothelial (EA.hy926) cells upon dengue virus (DENV) infection and TNF-α treatment identified 450 differentially altered proteins. Among them, decreased levels of moesin, actin stress fiber rearrangements, and dot-like formations of vinculin were observed with western blot analyses and/or immunofluorescence staining (IFA). In vitro vascular permeability assays using EA.hy926 cells, seeded on collagen-coated transwell inserts, showed low levels of transendothelial electrical resistance in treated cells. The synergistic effects of DENV infection and TNF-α treatment caused cellular permeability changes in EA.hy926 cells, which coincided with decreasing moesin levels and the production of abnormal organizations of actin stress fibers and vinculin. Functional studies demonstrated moesin overexpression restored transendothelial permeability in DENV/TNF-α-treated EA.hy926 cells. The present study improves the understanding of the disruption mechanisms of cytoskeleton proteins in enhancing vascular permeability during DENV infection and TNF-α treatment. The study also suggests that these disruption mechanisms are major factors contributing to vascular leakage in severe dengue patients.

## 1. Introduction

Dengue virus (DENV), a member of genus *Flavivirus* in the family of *Flaviviridae*, is transmitted to humans through the bites of infected *Aedes* mosquitoes. Dengue symptoms range from mild dengue fever (DF) to severe disease: dengue hemorrhagic fever (DHF) and dengue shock syndrome (DSS). Epidemiological studies have estimated that nearly 100 million cases of the DENV infections occur worldwide each year. The clinical manifestations of DHF and DSS include pleural effusion, ascites, and hypoproteinemia [1]. These forms are also associated with hypovolemic shock, vascular permeability, and plasma leakage. The DENV induces innate immune cells to secrete cytokines and chemokines. These inflammatory mediators mediate adaptive immune T cells and B cells, resulting in the release of cytokines and antibodies, respectively [2]. Dengue infection and/or an immune response mediated by cytokines or chemokines of activated lymphocytes leads to endothelial cells (ECs) becoming the target sites in dengue pathogenesis. ECs are the primary fluid barrier of the vasculature. Furthermore, cytotoxic granules, Fas, cytokines, and complement activation released from adaptive immune cells (especially tumor necrosis factor [TNF-α]) could induce signaling events and cause cytoskeleton changes that destabilize F-actin and increase EC permeability [2,3].

ECs play an important role in blood flow regulation and vascular biology. During a DENV infection, the EC barrier is destroyed, resulting in plasma leakage [3]. The DENV demonstrated the ability to infect ECs in a mouse model [4]. Moreover, in DENV-infected human ECs, the virus affected the expression levels of host proteins, especially vascular endothelial cadherin (VE-Cadherin), Zonula occludens-1 (ZO-1), and platelet-EC adhesion molecule-1 (PECAM-1) [5]. Another study showed that a high level of TNF-α was strongly correlated with the severity of dengue patients [6]. TNF-α is a pro-inflammatory cytokine produced by immunologically competent cells. It ordinarily mediates in the innate immune regulation and response as part of the host defense against infection. One of the interested characteristic of ECs as a source of TNF-α after they are stimulated with lipopolysaccharide (LPS) or interleukin-1alpha (IL-1alpha) [7]. The TNF-α secreted by ECs not only promoted distinct anatomical patterns and enhanced adhesion molecule expression in leukocytes, but also diminished and redistributed extra- and intracellular proteins [8]. The combination of TNF-α and IFN altered EC morphology by forming gap junctions, leading to actin rearrangement and increased vascular permeability [9]. A recent report documented that the synergistic effects of DENV2 infection and TNF-α treatment on human EC lines (EA.hy926 cells) caused adhesion molecule reorganization, which is a possible factor for vascular permeability [10]. It is essential to conduct studies on the alterations to proteomes in ECs during DENV infection and TNF-α treatment to understand the molecular mechanisms of vascular leakage in severe dengue infections. The present study investigated the alterations to cytoskeleton proteins and correlated the functional significance of those altered cytoskeleton proteins with the transendothelial permeability of human ECs upon DENV-2 infection and TNF-α treatment. The knowledge obtained from a functional study of cytoskeleton proteins will pave the way for understanding the mechanism of vascular leakage during DENV infections.

## 2. Materials and Methods

### 2.1. Dengue Virus and Monoclonal Antibodies

DENV2 strain 16681 and mouse monoclonal antibody against DENV 1–4 (4G2) were generously provided by the Armed Forces Research Institute of Medical Sciences, Bangkok, Thailand. 

### 2.2. Dengue Virus Propagation in C6/36 Cells

A large stock of DENV2 strain 16681 was used by this study. The strain was propagated in C6/36 cells. Briefly, monolayers of C6/36 cells were cultivated in a maintenance medium (L-15 medium; Gibco, Grand Island, NY, USA). The medium contained 10% FBS (Gibco); 10% tryptose phosphate broth (TPB; Sigma–Aldrich Corporation, St. Louis, MO, USA); 36 μg/mL penicillin (Sigma–Aldrich); and 60 μg/mL streptomycin (Sigma–Aldrich). DENV2 was added at a multiplicity of infection (MOI) of 0.01 at room temperature (RT) for 6 h, with gentle rotation. After 6 h, maintenance medium was added, and the DENV2 was further incubated at 28 °C. The virus culture supernatants were first harvested after a week of incubation, following which fresh medium was added. The infected cells were monitored for appearance of the cytopathic effect by regular harvesting of the virus supernatant until the infected cells fully exhibited the cytopathic effect. Cell debris was removed by centrifugation at 900× *g* for 5 min. The clarified supernatants were aliquoted and preserved as virus stocks at −70 °C until use.

### 2.3. Dengue Virus Titration

The culture supernatants from the DENV-infected cells were titrated in Vero cells using a focus forming assay. In brief, Vero cells were grown in 96-well tissue-culture plates for a day until confluence. The monolayer cells were cultivated with serial 10-fold dilutions of viral suspensions in minimum essential medium containing 2% FBS, 2 mM l-glutamine, 36 μg/mL penicillin and 60 μg/mL streptomycin for 2 h in a humidified atmosphere incubator with 5% CO_2_ at 37 °C. Thereafter, overlay medium containing 2% FBS, 1.5% carboxymethyl cellulose (CMC; Sigma–Aldrich), 2 mM l-glutamine, 36 μg/mL penicillin, and 60 μg/mL streptomycin were added to each well (100 μL/well). The infected cells were incubated in a humidified atmosphere incubator with 5% CO_2_ at 37 °C for 3 days. After removal of the medium from the DENV-infected cells, the adherent cells were washed 3 times with phosphate buffered saline (PBS; pH 7.4) and fixed with 3.7% formaldehyde in PBS at RT for 10 min. In the permeabilization step, 1% Triton X-100 was added for 10 min. The cells were subsequently incubated with mouse anti-DENV envelope monoclonal antibody (clone 4G2) at 37 °C for 1 h. This was followed by the addition of horseradish peroxidase-conjugated rabbit anti-mouse immunoglobulins (Dako; Santa Clara, CA, USA). A dilution of 1:1000 was used in PBS containing 2% FBS and 0.05% Tween-20. The process was undertaken in the dark at 37 °C for 30 min. To develop an enzymatic reaction, the adherent cells were incubated with a substrate solution containing 0.6 mg/mL diaminobenzidine, 0.03% H_2_O_2_, and 0.08% NiCl_2_ in PBS at RT in the dark for 5 min. After washing three times with PBS, the dark brown foci of the DENV-infected cells were counted under a light microscope and calculated for virus titer. The titer of virus stock was reported as focus-forming units (FFU)/mL.

### 2.4. DENV Infection and/or TNF-α Induction in EA.hy926 Cells

The human umbilical vein cell line, EA.hy926 cells used in the study is derived from the establishment of the fusion of primary human umbilical vein cells with a thioguanine-resistant clone of human lung carcinoma epithelial cells (A549). This EA.hy926 cells have characteristics of differentiated endothelial cell functions, such as angiogenesis, homeostasis/thrombosis, and inflammation [11,12]. EA.hy926 cells were cultivated in Dulbecco’s modified Eagle’s medium (DMEM)/F 12 medium containing 10%FBS, 36 μg/mL penicillin, and 60 μg/mL streptomycin and used as an in vitro model for the previous study of paracellular and transcellular permeability because they have characteristics similar to human endothelial cells [10,13]. EA.hy926 cells were seeded on each well of a six-well tissue-culture plate for 24 h. After overnight incubation, DENV2, at an MOI of 5, was added to a confluent monolayer of EA.hy926 cells in DMEM/F-12 medium containing 2% FBS, 36 μg/mL penicillin, and 60 μg/mL streptomycin. The conditions were maintained in a humidified atmosphere incubator with 5% CO_2_ at 37 °C for 2 h. Thereafter, 1 ng/mL TNF-α was added into TNF-α conditions. In addition, EA.hy926 cells without virus infection and TNF-α induction were cultured in parallel and used as the mock control. After incubation at 37 °C for 2 h, the virus supernatants were replaced with 2% FBS in DMEM/F-12. The cultured supernatants and cells were harvested at 24, 36, and 48 h post-infection.

### 2.5. Analysis of Viral Infection by Flow Cytometry

To determine the viral infection, the mock-infection, DENV2-infection, and/or TNF-α induction conditions were detected for dengue viral antigen. The adherent cells were trypsinized with 0.1% trypsin in 2.5 mM EDTA in PBS, and then resuspended in growth medium. The cells were subsequently washed with PBS by centrifugation at 2500× *g*, at 4 °C, for 2 min. After harvesting, the cells were fixed with 2% formaldehyde in PBS at RT for 20 min. The cells were permeabilized with 0.1% triton X-100 in PBS at RT for 10 min, followed by the addition of mouse anti-DENV E monoclonal antibody (clone 4G2) at 37 °C for 1 h. Next, EA.hy926 cells were incubated with Alexa 488 rabbit anti-mouse immunoglobulins (Jackson ImmunoResearch Laboratories, Inc., West Grove, PA, USA) at a dilution of 1:50 in 1% FBS and 0.1% triton-X 100 in PBS at 37 °C for 30 min. The cells were suspended in PBS before being analyzed with FACScan (BD Biosciences, SanJose, CA, USA).

### 2.6. Analysis of Cell Viability Using Flow Cytometry

The percentage of cell viability, the mock-infections, DENV2 infections, and/or TNF-α induction were measured using flow cytometry. Briefly, cells were harvested from each treated condition. EA.hy926 cells were washed and resuspended in Annexin V binding buffer, twice. The cells were then incubated with Annexin V-FITC (ImmunoTools, Friesoythe, Germany) for 15 min in the dark. Annexin V binding buffer and Propidium Iodine (ImmunoTools) were added and immediately measured by flow cytometry.

### 2.7. Analysis of Cytoskeleton Protein Fraction by Mass Spectrometry

#### 2.7.1. Isolation of Cytoskeleton Proteins

Cytoskeleton proteins were isolated using ProteoExtract Cytoskeleton Enrichment and Isolation Kit (EMD Millipore, MA, USA) according to the manufacturer’s protocol. Briefly, adherent cells were washed with PBS twice after removing the media. Cellular extraction buffer was added and incubated for 90 s and collected as cytosolic fraction. Thereafter, cytoskeleton wash buffer was added and collected as cytosolic fraction. Nuclear extraction buffer was then added, and the buffer was gently collected as nuclear fraction. Subsequently, EA.hy926 cells were washed once with cellular extraction buffer and washed twice with cytoskeleton wash buffer. Cytoskeleton solubilization buffer was then added on the cell surface. Adherent cells were scraped using a cell scraper. Lastly, the samples were sonicated and continuously centrifuged at 13,000× *g* to remove debris. The cytoskeleton protein fraction was kept at −20 °C until use.

#### 2.7.2. Determination of Protein Concentration by Bradford Technique

To measure the concentration of protein, a Bradford dye-binding protein assay was performed. First, to establish a standard curve, 2 mg/mL of BSA was diluted in sterilized deionized water. Both standard and samples were added to Quick Start Bradford 1x dye reagent (Bio-Rad, Hercules, CA, USA) and incubated at RT for 5 min. The dye-bound standard and samples were then measured for absorbance at a wavelength of 595 nm by a UV-160A spectrophotometer (Shimadzu, Kyoto, Japan) within 20 min of the completion of the incubation. The absorbance for each concentration of standard protein was then plotted to give the standard curve. The curve was used to calculate the concentrations of the samples. The protein lysate was stored at −20 °C until use.

### 2.8. Separation of Protein by First Dimension-Polyacrylamide Gel Electrophoresis (1D-PAGE)

Thirty micrograms of total proteins were mixed with 2× sample buffer and loaded into 12% polyacrylamide gel before electrophoresis at 150 volts for 2 h. The separating proteins were stained with Coomassie Brilliant Blue R-250 for 10 min and subsequently washed twice with destaining solution at RT for 20 min. The gel was stored overnight in destaining solution at 4 °C.

### 2.9. In-Gel Tryptic Digestion

The gel was manually cut into pieces and washed with 100 µL of 50 mM ammonium bicarbonate. After washing, the solution was removed, and the gel pieces were destained with 500 µL of 50 mM ammonium bicarbonate and 500 µL of acetonitrile. Destaining was performed at 4 °C overnight or until the gel pieces were cleared. Thereafter, solution was removed from the gel pieces. Disulfide bonds were reduced by adding 100 µL of 4 mM DTT in 50 mM ammonium bicarbonate to the gel pieces, and incubating at 60 °C for 15 min. Then, the solution was left to cool to RT before adding 7 µL of 250 mM α-Iodoacetamide for alkylation. Incubation was carried out at RT in the dark for 30 min. The solution was quenched by adding 3 µL of 4 mM DTT in 50 mM ammonium bicarbonate. The gel pieces were rehydrated by 100% CAN for 20 min, after which all solution was removed. The gels were left until completely dried. The peptides were digested from the gel pieces by adding 10 µL of 20 µg/200 µL trypsin in 50 mM ammonium bicarbonate. About 100 µL of 50 mM ammonium bicarbonate and 100 µL of 5% acetonitrile in 50 mM ammonium bicarbonate were added to cover the gel pieces. They were incubated at 37 °C for at least 4 h or overnight. Peptide solutions were subsequently added with 200 µL of acetonitrile and incubated at RT for 20 min. After centrifugation, the supernatants were collected into 1.5-mL microcentrifuge tubes. The peptides were finally concentrated by a Savant SpeedVac Concentrator (Thermo Fisher Scientific, Waltham, MA, USA). The peptide pellets were resuspended in 75 µL of 0.1% formic acid. About 15 µL of peptide solution was loaded into tubes for mass spectrometry.

### 2.10. Analysis of Peptides by Mass Spectrometry and Protein Identification

After tryptic digestion, the peptides were extracted from the gel and further subjected to an Ultimate 3000 nano-LC system (Dionex, Surrey, UK). The eluted peptides were directly analyzed by MicroToF Q II mass spectrometer (Bruker Daltonik, Bremen, Germany). The LC-MS/MS raw data files were processed and converted into mascot generic format files using Data Analysis software (version 3.4; Bruker Daltonik). The files were searched using Mascot (version 2.4.1; Matrix Science, London, UK) against the National Center for Biotechnology Information database. Protein hits from the MASCOT search with a minimum of at least two peptides and a minimum score of 20 were filtered. Only proteins with a confidence level of 95% were reported. For protein semiquantification, the exponentially modified protein abundance index (emPAI) was applied.

### 2.11. Western Blot Analysis

The mock-infected, DENV2-infected, and/or TNF-α induction EA.hy926 cell samples used in the experiment were prepared and analyzed by western blot. To determine if the altered proteins had a higher or lower level of protein response, the protein lysate was prepared from these conditions by using a radioimmunoprecipitation assay (RIPA) buffer, containing phosphatase and protease inhibitors, for 1 h. Then, 40 µg of extracted proteins were completely mixed with 4× loading buffer containing 50 mM Tris-HCl (pH 6.8), 2% SDS, 0.1% bromophenol blue, and 10% glycerol. The solution was heated at 95 °C for 5 min before being subjected to 8% SDS-PAGE. Electrophoresis was carried out in 1x running buffer at a constant 150 volts for 90 min. Subsequently, proteins were transferred to nitrocellulose membranes using wet-tank transferring apparatus (Mini Trans-Blot Cell; Bio-Rad). Electroblotting was carried out overnight at 20 volts, 80 watts, and 400 mA. Nonspecific binding proteins were blocked using 5% skimmed milk in Tris-HCl, 0.1% Tween 20 (TBST) for 1 h. This was followed by overnight incubation with primary antibody in 5% skimmed milk in TBST at 4 °C. After washing three times with TBST, the membrane was incubated with horseradish peroxidase-conjugated secondary antibody (DAKO; Santa Clara, CA, USA) in 5% skimmed milk in TBST at a dilution of 1:1000. This was conducted at RT in the dark for 1 h, and it was followed by three further washes with TBST. Then, protein bands were visualized by using an enhanced chemiluminescence detection kit (Luminata Forte Western HRP substrate; EMD Millipore). After drying, the membrane was inserted into an X-ray film cassette to expose its signal to the X-ray film. Dark bands of protein were observed after developing the photographic film. In order to assess the different levels of protein, the intensities of the protein bands of the mock-infected and DENV2-infected samples were compared. The relative expression levels of the proteins were evaluated by normalization of their protein band intensities to glyceraldehyde 3-phosphate dehydrogenase (GAPDH) intensity by using ImageJ software (National Institutes of Health, Bethesda, MD, USA). Data are displayed as mean ± SE. The Mann–Whitney test and ANOVA or Kruskal–Wallis one-way ANOVA were used for the analyses of the test and control groups. A *p* value lower than 0.05 was deemed significant.

### 2.12. Immunofluorescence Staining

To determine the localization of proteins, EA.hy926 cells were fixed in 4% paraformaldehyde in PBS at RT for 20 min. After washing twice with PBS, cells were permeabilized with 1% Triton X-100 in PBS at RT for 10 min. Then, the cells were incubated with primary antibody in 1% BSA in PBS at a dilution of 1:50 at 37 °C for 1 h. Next, the excess primary antibody was washed three times with PBS. The cells were incubated with 0.5xX Phalloidin-Tetramethylrhodamine Conjugate (Santa Cruz Biotech, Dallas, TX, USA) and/or 1:1000 dilution of chicken anti-mouse Alexa Fluor 647 Conjugate (Invitrogen, Carlsbad, CA, USA) and donkey anti-rabbit Alexa Fluor 488 Conjugate (Invitrogen, Carlsbad, CA, USA). Hoechst 33258 dye (Molecular Probes, Eugene, OR, USA) was used to stain the nucleus in 2% FBS in PBS at RT in the dark for 1 h. After washing off the excess second antibody three times with PBS, each glass cover slip was mounted with 50% glycerol in PBS and sealed on the slide with nail polish. The samples were visualized under laser confocal microscope (LSM 800 with Airyscan; Carl Zeiss, Jena, Germany).

### 2.13. In Vitro Vascular Permeability Assay

To determine the cellular permeability changes with DENV2 infection and/or TNF-α induction using transepithelial electrical resistance (TEER) measurement, EA.hy926 cells were seeded into transwell inserts on the upper well that were collagen-coated with a transparent polyethylene terephthalate membrane (EMD Millipore). Confluent EA.hy926 cells were infected with DENV2 at a MOI of five and/or induced with 1 ng/mL of TNF-α. To measure the membrane potential and resistance of the ECs in culture 24 h post infection, a Millicell ERS (Electrical Resistance System)-2 Voltohmmeter (EMD Millipore) was used to qualitatively measure cell monolayer health and quantitatively measure cell confluence. Between the sample measurements, DMEM/F-12 was used to wash the silver/silver chloride electrode tip to avoid cross contamination. The results were calculated as resistance over the area according to Ohm’s law.

### 2.14. Transient Transfection of Recombinant Moesin Expression Plasmid in EA.hy926 Cells

EA.hy926, 1 × 10^5^ cells were seeded into a 24-well plate until the cell density reached 70% to 80% confluence. Transfection with either pCMV6 or pCMV6-moesin plasmid was performed using the Targefect F2 reagent plus Virofect enhancer (Targeting Systems, El Cajon, CA, USA). Briefly, 0.625 μg of plasmid DNA was mixed with 1.2 μL of Targefect-HUVEC and 2.5 μL of Virofect in 62.5 μL of high-glucose DMEM, and then incubated at 37 ℃ for 25 min to form a transfection complex. Thereafter, the complex was added to a 250 μL DMEM/F12, 10% FBS medium. After the transfection process, EA.hy926 was incubated at 37 °C in a humidified CO_2_ incubator for 24 h. The assay for protein production was performed at 24 h posttransfection.

### 2.15. Statistical Analysis

At least three independent experiments were done. They are reported as mean ± SE. Statistical differences between the groups were analyzed with an unpaired *t*-test using GraphPad Prism software (version 7.0; GraphPad Software, La Jolla, CA, USA). A *p* value < 0.05 was considered as a statistically significant difference between groups.

## 3. Results

### 3.1. Infection and Cell Viability in DENV2-Infected EA.hy926 Cells

#### 3.1.1. DENV Infection

To determine the percentage of DENV2 infection, DENV E (envelope) protein, one of the structural proteins of DENV, was detected as the marker of infection by intracellular staining. It was subsequently analyzed by flow cytometry. EA.hy926 cells were harvested and stained with a monoclonal antibody against dengue E protein (clone 4G2). At 24 h post-infection, the percentages of infection at an MOI of 5 were 1.79%, 57.40%, 1.98%, and 60.08% for the mock-infected, DENV2-infected, TNF-α, and TNF-α plus DENV2 treated cells, respectively. By 36 h post-infection, the percentages had decreased to 2.08%, 32.47%, 1.22%, and 26.05%, respectively. Finally, by 48 h post-infection, the percentages of infection had declined to 0.14%, 11.17%, 0.28%, and 10.14%, respectively (Supporting Information S1A).

#### 3.1.2. The Percentage of Cell Death

To clarify the process of cell death, double staining of annexin V/FITC and PI was analyzed by flow cytometry. The percentages of cell death for the mock-infected, DENV2-infected, TNF-α, and TNF-α plus DENV2 treated cells were compared at various incubation times (Supporting Information S1B). At 24 h post-infection, there were no significant differences in the percentages of cell death for each treatment condition. The percentages were 7.73%, 8.30%, 4.81%, and 9.40% for the mock-infected, DENV2-infected, TNF-α, and TNF-α plus DENV2 treated cells, respectively. This indicated that there were low levels of cell death at 24 h post infection. However, at 36 h post infection, high percentages of cell death were observed for the DENV2-infected (43.61%) and the TNF-α plus DENV2 (48.01%) treated cells. By contrast, the values for the mock-infected and the TNF-α treated cells were much lower (6.56% and 7.03%, respectively). At 48 h post infection, high percentages of cell death were again found for the DENV2-infected and the TNF-α plus DENV2 treated cells, with 39.72% and 67.58%, respectively. Only 11.92% of cell death was observed for the mock-infected cells and 10.08% for the TNF-α cells. The results showed a clear decline in the viability of the infected cells over time.

### 3.2. Proteomic Study of Cytoskeleton Protein Fraction Isolated from DENV2 and TNF-α-Treated EA.hy926 Cells

The aim of this experiment was to determine the expression of cytoskeleton proteins after DENV2 and TNF-α treatment, compared with the mock control, by mass spectrometry.

#### 3.2.1. Confirmation of the Purity of Cytoskeleton Protein Fraction Isolated from EA.hy926 Cells

Cytoskeleton protein fraction was isolated from DENV2- and TNF-α-treated EA.hy926 cells using ProteoExtract Cytoskeleton Enrichment and Isolation Kit according to the manufacturer’s protocol. There were three fractions: soluble, nuclear, and cytoskeleton. To check the purity of isolation, the mock cells were used to examine the protein of each representative marker of fraction. After isolation, proteins from the soluble, nuclear and cytoskeleton fractions were analyzed using SDS-PAGE. Furthermore, the purity of proteins was continuously determined by western blot (Supporting Information S2). The cytoskeleton protein vimentin was used to indicate cytoskeleton protein, while GAPDH was the marker for cytosolic protein. The results revealed that vimentin was found only in the cytoskeleton fraction. This suggests that the cytoskeleton protein had been completely extracted from the EA.hy926 cells.

#### 3.2.2. Alterations of Proteome in DENV2- and TNF-α Treated-EA.hy926 Cells

Cytoskeleton proteins of both the mock control and treated cells were isolated and separated by SDS-PAGE. Protein bands from both groups were detected by staining with Coomassie Brilliant Blue and mass spectrometry. The mass spectrometry analysis of the cytoskeleton protein fractions revealed 450 altered proteins in the 2 sample groups (Supporting Information S3). These altered proteins were classified using the Panther classification system, based on molecular function. Of the altered proteins, 30.6% were structural molecules, transporters, and receptors, while 26.4% were involved in antioxidant, catalytic, signal-transduction, and translation-regulator activities. The remainder (43%) were nucleic acid binding proteins, enzyme modulator proteins, and others (Table 1). Some cytoskeleton proteins had multiple protein classes. One protein had various functions.

**Figure 1:**
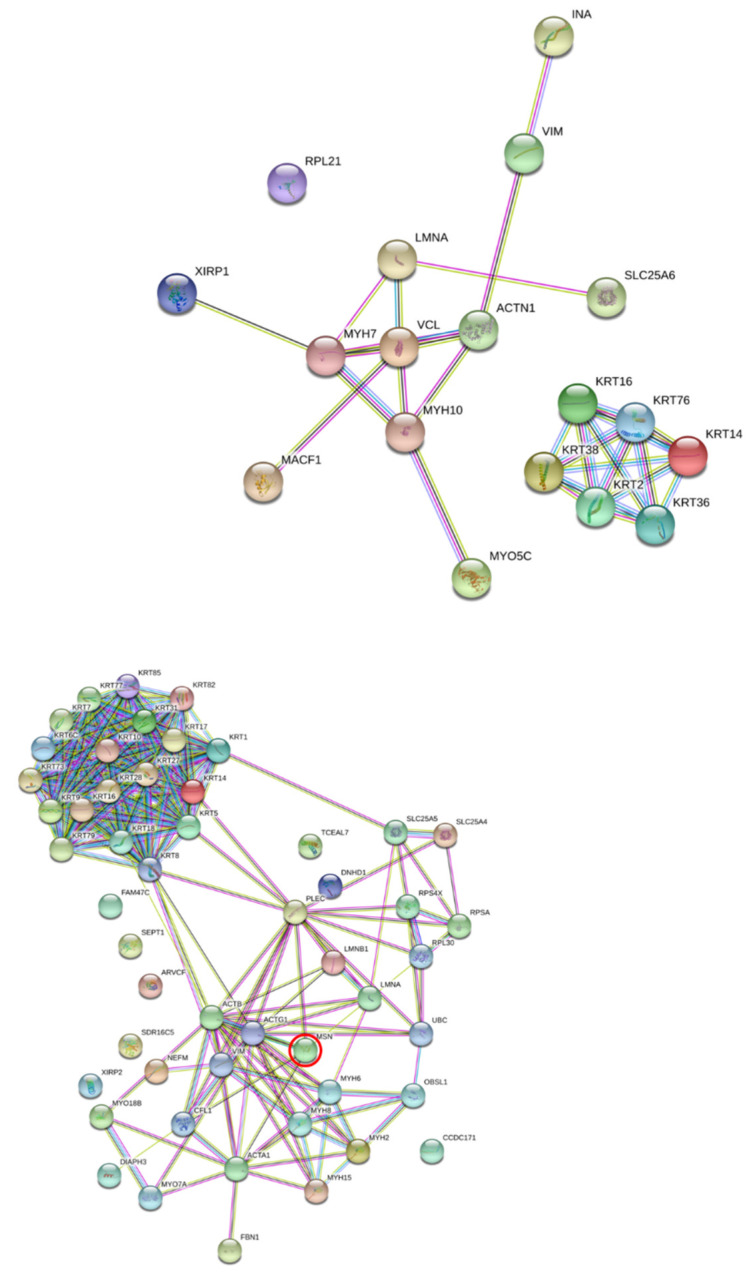
Analysis of global protein network interaction using STRING software. Interaction of protein network in altered proteins from EA.hy926 cells during treatment with DENV2 and TNF-α was analyzed by STRING software. The downregulated proteins were found to be significantly associated with, and to interact with, cytoskeleton proteins. Actin was one of the priority proteins whose functions and association with membrane permeability were focused on. Accordingly, actin and moesin were highlighted from the global protein interactions network. The connecting lines between the protein nodes indicate protein-protein interactions.

After classification, the major proteins were structural and cytoskeletal proteins (Supporting Information S4). These were further analyzed by global protein network analysis using STRING software. To address the significance function of the altered proteins, a number of differentially expressed proteins were identified in the DENV2- and TNF-α-treated EA.hy926 cells. This study aimed to investigate the functions of cytoskeletons that play a role in transendothelial permeability. The altered cytoskeleton proteins were analyzed by STRING software (Figure 1). The downregulated proteins were found to have significant associations and interactions with the cytoskeleton proteins. Actin was one of the priority proteins to focus attention on their functions in association with membrane permeability. Accordingly, actin and moesin were highlighted in the global protein interactions network.

### 3.3. Confirmation of the Proteomes Data

Among the altered proteins from the mass spectrometry analysis, moesin, β-actin, and vinculin were validated to confirm the protein expression levels in the DENV2-infected and/or TNF-α-induced EA.hy926 cells. Western blot analysis and immunofluorescence staining were used.

#### 3.3.1. Analysis of Altered Proteins by Western Blot Analysis

**Figure 2:**
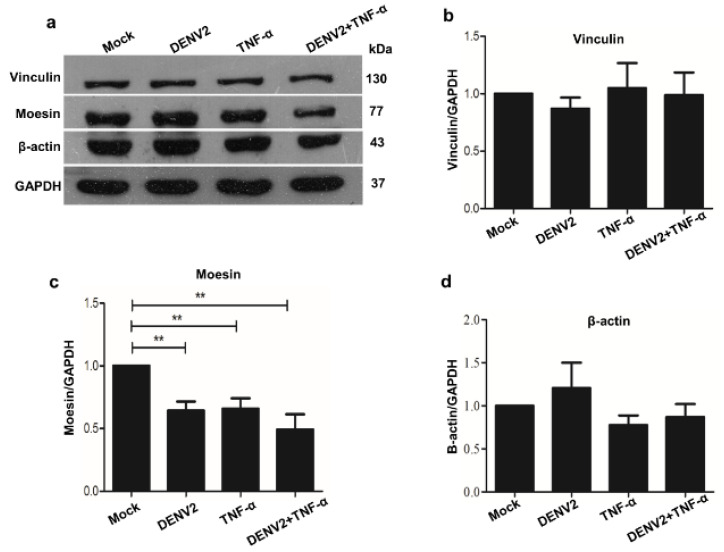
Western blot analysis of altered cytoskeleton proteins in EA.hy926 cells after 24 h of treatment with DENV2 and TNF-α. Forty micrograms of total proteins isolated from each group was run on 12% SDS-PAGE, and then transferred onto nitrocellulose using a wet tank chamber. Western blot analysis was performed using specific antibody against moesin, actin, and vinculin, with antibody against GAPDH being used as the internal loading control (**a**). Quantitative analysis of vinculin, moesin, and actin in EA.hy926 cells (**b**–**d**). A *p* value < 0.05 indicates statistical significance, as follows: ** *p* < 0.005.

Forty micrograms of protein lysate were separated and analyzed by SDS-PAGE and western blot. Relative to the mock control cells, the protein level of moesin was decreased in DENV2 infection, TNF-α induction, and combination treatment of DENV2 infection and TNF-α induction cells. However, β-actin and vinculin showed no significant changes in protein level for all treatment conditions at 24 h post-infection, compared with the mock control cells. This suggests that a decrease in the quantity of moesin only occurred during DENV2 infection and/or TNF-α induction (Figure 2).

#### 3.3.2. Localization of Altered Proteins Using Immunofluorescence Staining

**Figure 3:**
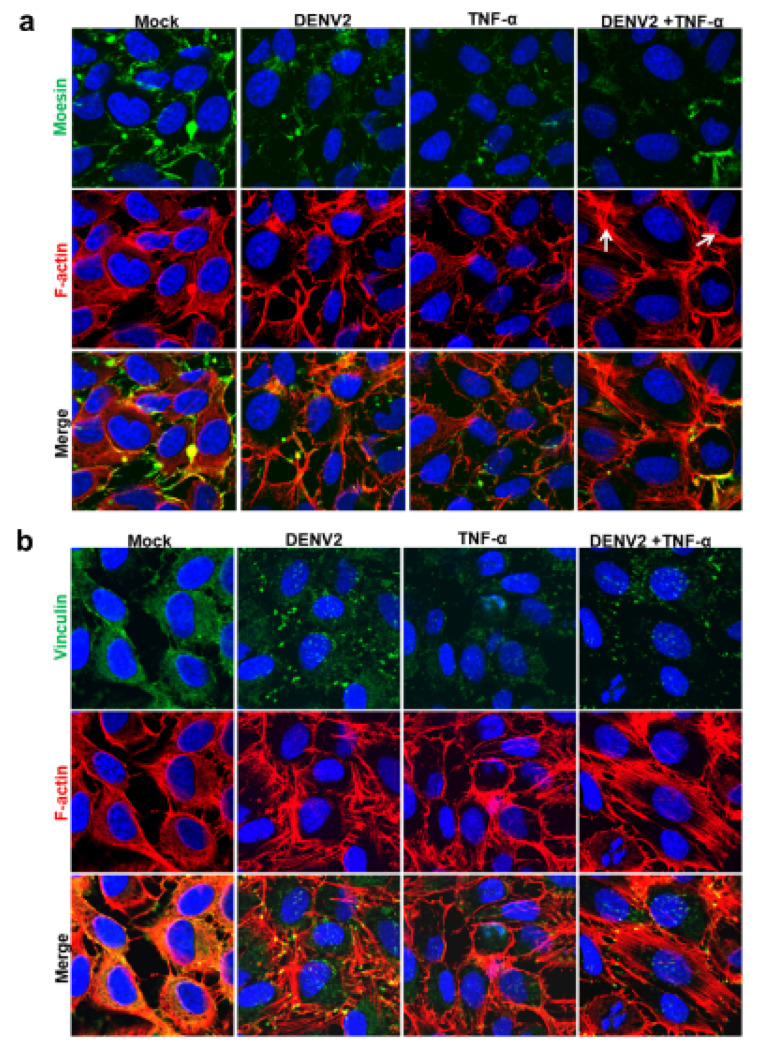
Immunofluorescence staining of moesin (green) and F-actin (red) (**a**), and of vinculin (green) and F-actin (red) (**b**), in DENV2-, TNF-α- and DENV2-plus-TNF-α-treated EA.hy926 cells after 24 h of incubation. Hoechst 33258 was used to stain the nucleus (blue). Fluorescent images were captured by a laser confocal microscope (Zeiss LSM 800 with Airyscan) at 630 magnification.

EA.hy926 cells on a glass cover slip were stained with specific antibody and observed under a confocal laser microscope (Figure 3a). The moesin in the DENV2-infected and/or TNF-α-induced EA.hy926 cells was found to be deteriorated, especially with the synergistic effect of DENV2 and TNF-α. This result correlates with the data of the Western blot analysis. Phalloidin staining showed F-actin stress fibers in the DENV-infected and/or TNF-α-treated EA.hy926 cells (white arrow in Figure 3a). The synergistic effect revealed distinct alterations of the cytoskeleton proteins; this may be a possible cause of permeability changes. Moreover, vinculin staining showed a condensed, dot-like structure in the DENV2-infected, TNF-α, and DENV2 plus TNF-α treated cells. This contrasted with a uniform localized pattern in the cytoplasm of the mock control cells (Figure 3b).

### 3.4. Determination of Functional Significance of EA.hy926 Cells during DENV2 Infection and/or TNF-α Treatment

#### 3.4.1. In Vitro Permeability Assay

**Figure 4:**
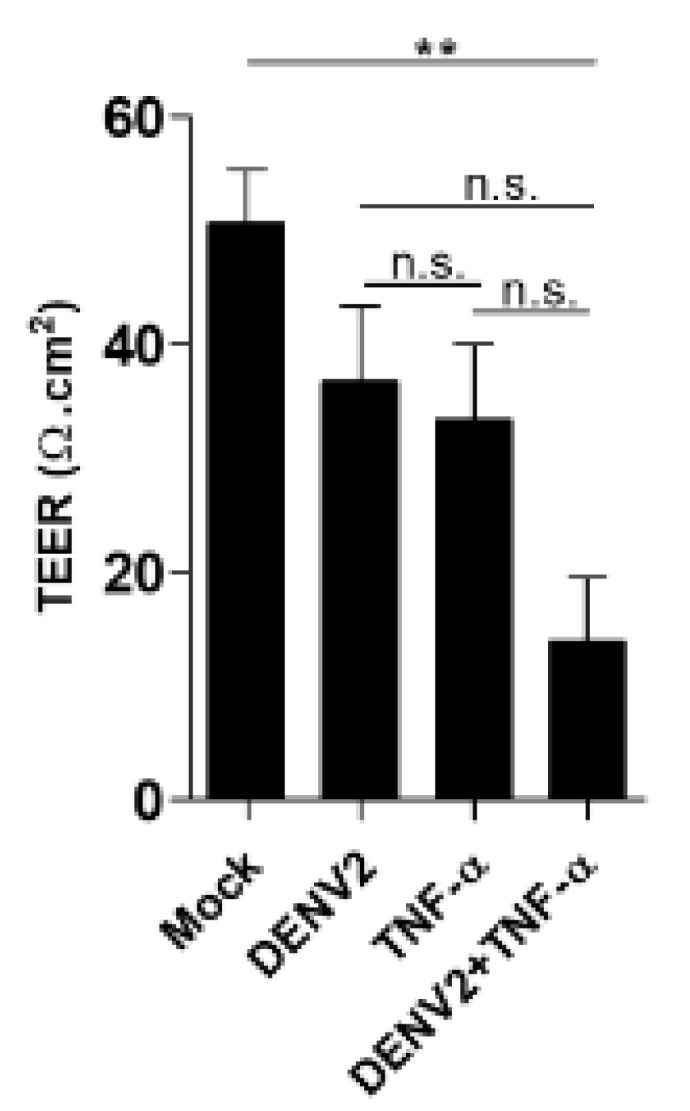
In vitro vascular permeability assay was performed in EA.hy926 cells. The result of 3 TEER experiments in mock, DENV2, TNF-α, and DENV2 plus TNF-α are shown. A *p* value < 0.05 indicates statistical significance, as follows: ** *p* < 0.005.

To determine the synergistic effect of DENV2 infection and TNF-α treatment by TEER, EA.hy926 cells were infected with DENV2 and/or treated with TNF-α for 24 h. The endothelial permeability of monolayer cells on transwell inserts was then evaluated by Millicell ERS-2 Volt-Ohm Meter. The DENV2-infected combined with TNF-α-treated monolayer cells revealed a significant increase in endothelial permeability, evidenced by a low TEER value. In comparison, the DENV2 infection or TNF-α treatment alone cells showed only a slight decrease in their endothelial permeability, compared with the mock control cells (Figure 4).

#### 3.4.2. Transendothelial Permeability could Be Restored during Overexpression of Moesin

**Figure 5:**
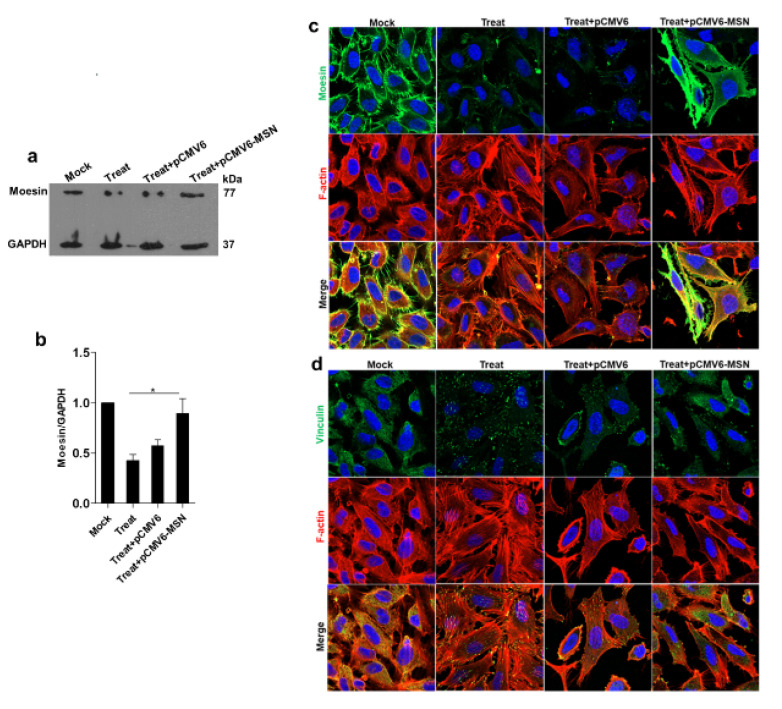
Analysis of moesin overexpression in DENV2 plus TNF-α-treated EA.hy926 cells. A higher moesin level was observed in EA.hy926 cells transfected with recombinant moesin plasmid than in cells transfected with empty plasmid. The intensity of moesin was determined by Western blot (**a**,**b**). The localizations of moesin, vinculin, and F-actin are also demonstrated (**c**,**d**), * *p* < 0.05.

**Figure 6:**
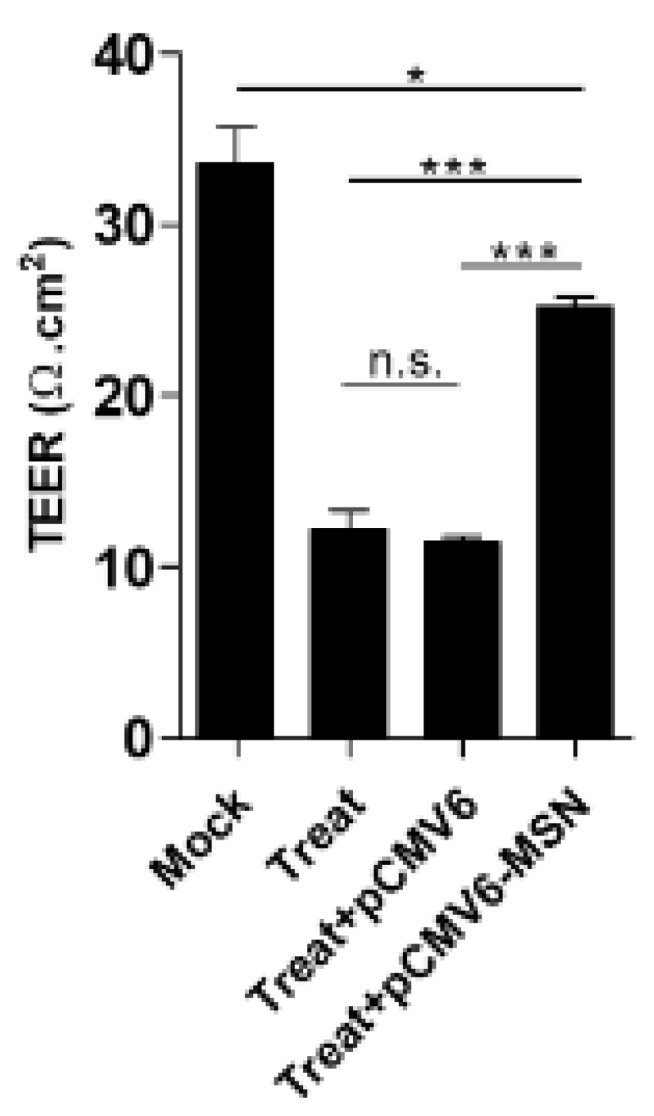
An in vitro vascular permeability assay was performed in DENV2-plus-TNF-α-treated EA.hy926 cells. The TEER value decreased in the treated cells, whereas the TEER level was restored in cells transfected with only recombinant moesin plasmid, * *p* < 0.05 and *** *p* < 0.001.

The functional effect of moesin in response to maintaining transendothelial permeability was assessed using overexpression of moesin in DENV2 infection and TNF-α treatment. The moesin was shown to be higher expressed. This was demonstrated by a Western blot analysis of treated EA.hy926 cells transfected with only recombinant moesin plasmid, compared with treated cells transfected with empty plasmid (Figure 5). The immunofluorescence staining also showed a higher expression of moesin in the same manner as the Western blot results. The TEER value was significantly restored in cells transfected with recombinant moesin plasmid (Figure 6).

## 4. Discussion

One of the distinguishing clinical manifestations of DENV infections is plasma leakage. This has been observed to increase vascular permeability in cases of DHF and DSS. The DENV infection and the high secretion of cytokines or chemokines lead to significant plasma leakage in tissue spaces, resulting in profound shock and life-threatening disease (DHF and DSS). The cytoskeleton works as a vehicle to transport the viral protein into the nucleus and plays a role during adenovirus and herpes simplex virus infections [14]. Cytoskeleton reorganization changes the steady-state distribution of the membrane organelles in order to maintain their functions [15]. Viral RNA replication takes place in replication complexes. These are composed of viral RNA and proteins, and they are formed on the endoplasmic reticulum membrane. This membrane structure is where viral RNA translation, protein processing, and virion assembly take place [16]. The dynamics of cytoskeletal and cytoskeleton-associated proteins are important components of the regulation of the endothelial barrier function. The actin cytoskeleton links to the tails of junction and adhesive proteins as well as extracellular matrix proteins. Moreover, actin is significant in the stabilization of the intercellular junctions and the maintenance of endothelium integrity [17]. A previous study reported that the synergistic effects of DENV infection and TNF-α induction leads to altered cellular permeability and vascular leakage [10,18]. In that work, human EC lines (EA.hy926) were used as a model for vascular leakage experiments to investigate the effects of cytoskeleton proteins on DENV infection and TNF-α induction. DENV abolished host cells by invading cells through cytoskeleton proteins. In the secondary DENV infection patients, TNF-α was found in serum. The plasma leakage in severe DENV patients was caused not only by the DENV infection, but also by cytokines secreted from immune cells.

In the current research, the isolation of cytoskeleton proteins was performed successively using the monitoring of protein marker of vimentin for cytoskeletal protein, with a high purity observed solely in the cytoskeleton fraction. The mass spectrometry and mascot search data revealed that more than 60% of proteins were structural, transporter, receptor, and binding proteins. The cytoskeletal proteins were studied further because of the prominent protein-protein interaction network. The western blot analysis also demonstrated a decrease in the level of the cytoskeletal protein moesin during DENV2 infection and TNF-α treatment. This reduction may play an important role in cellular permeability during the pathogenesis of a dengue infection. Moreover, the immunofluorescence staining revealed different patterns for the cytoskeletal proteins moesin, F-actin, and vinculin in human ECs.

Mass spectrometry analysis identifies the corresponding protein in their locality, which is a technique for identifying the specific proteins present in cells. The technique has also been utilized to compare posttranslational modifications in proteins. The altered proteins identified in the current study were mostly downregulated proteins. However, significant interactions were observed when the altered structural proteins were analyzed, especially among the downregulated proteins.

Usually, cytoskeleton proteins play an important role in maintaining the cellular permeability of cells. In DENV-infected patients, cytoskeleton proteins on ECs have been suggested to be dysfunctional and responsible for an imbalance in the control of vascular leakage. The maintenance of membrane integrity depends on both the normal expression of proteins and the quality of the protein localization.

Consequently, cytoskeleton proteins were investigated for functional linkage using STRING software. Of the altered cytoskeleton proteins, actin, moesin, and vinculin were chosen for validation in the study. The Western blot analysis revealed significant decreases only in moesin in the DENV2-infected and TNF-α treated-EA.hy926 cells, which correlated with the immunofluorescence assay results. Actin and vinculin proteins’ expressions were not significantly changed. This contrasts with DENV-infected HEK293T cells, in which the actin protein level was found to be significantly reduced during DENV infection at 24 h [19]. Moreover, in DENV-2-infected and/or TNF-α-treated EA.hy926 cells, F-actin rearrangements were observed, and they became actin stress fibers. Nevertheless, DENV-infected HEK293T cells displayed no actin reformation. In view of the results, cell-type specific differences may be a factor in cytoskeleton reorganization. The immunofluorescence staining results also revealed changes in the vinculin patterns of DENV2-infected and TNF-α-treated EA.hy926 cells. After DENV2 infection and TNF-α treatment, the vinculin protein was observed to take on a dot-like pattern.

The junctional complex in adjacent cells plays an essential role in maintaining the permeability of ECs. The cytoskeleton proteins in ECs are key to controlling the integrity of the endothelial barrier function. These proteins regulate the shape and motility of the cells. Viruses use the host cellular machinery to achieve their life cycle, and a number of cellular components are involved in the virus-cell interactions. In addition, cytoskeleton proteins provide a physical barrier to virus penetration and endocytic vesicles.

Moesin, vinculin, and actin cytoskeletons are host cell proteins which are disrupted during an infection via a diverse set of viral pathogens. Actin structure is required for the formation of the linear and continuous adherence junctions that are necessary for the maintenance of the barrier function of the ECs.

Moesin belongs to the ERM (Ezrin/Radizin/Moesin) family. It serves as a bridge between actin filaments and the plasma membrane. Moesin, an actin-binding protein, plays a role in cytoskeletal changes and paracellular gap formation. Although the moesin structure has an identical primary sequence homology to that of ezrin, it has a partially different function. Moesin is mainly expressed in ECs. Moreover, the phosphorylation of threonine residues at T558 activates moesin. The linker effects of moesin modify the cell morphology, motility, adhesion, mitosis, and polarity [20]. DENV-infected and TNF-α-induced EA.hy926 cells altered the moesin expression and actin reorganization.

After DENV infection and/or TNF-α treatment, cytoskeleton proteins at the cell-cell junction were pulled and formed discontinuous cell-cell junctions, leading to the creation of an intercellular gap. This study demonstrated that the synergistic effect between DENV2 infection and TNF-α induction could demolish moesin expression, redistribute vinculin, and reorganize the actin cytoskeleton during virus infection. We also demonstrated that moesin overexpression in treated EA.hy926 cells restored the function of transendothelial permeability, while the cells transfected with empty plasmid did not show any recovery effect. The result of recovery effect by moesin overexpression did not cause more cell death found in the experiment.

Another aspect of moesin relates to the binding of high-mobility group box 1 (HMGB-1) or the advanced glycation end product (AGE) to receptors to generate advanced glycation end-products (RAGE). This has been found to mediate moesin 558 threonine phosphorylation via the RhoA/ROCK and p38MAPK signaling pathways in HUVEC. This effect leads to F-actin rearrangement and hyperpermeability [20,21]. Furthermore, TNF-α, which are pro-inflammatory cytokines, induced the threonine phosphorylation of ERM, resulting in cytoskeleton changes in endothelial permeability in primary human pulmonary microvascular ECs [22].

Vinculin is a membrane-cytoskeletal protein that is involved in the linkage of the integrin adhesion molecule to the actin cytoskeleton. Vinculin was found to mediate cell adhesion remodeling and EC permeability in thrombin and oxidized phospholipid (oxidized 1-palmitoyl-2-arachido-noyl-*sn*-glycero-3-phosphocholine; OxPAPC) induced human pulmonary artery ECs [23,24,25]. Thrombin treatment caused actin stress fibers to attach to the focal adhesions. However, OxPAPC stimulated the formation of a diffuse peripheral actin rim and attached to peripherally localized focal adhesions.

The three binding sites of talin are important for the coupling of integrins to F-actin. Those binding sites also bind tightly to vinculin [26]. In certain conditions, talin may be an effective activator of vinculin. Rearrangement of the actin cytoskeleton depends on its tight connection to the plasma membrane. Phosphatidylinositol 4,5-bisphosphate is thought to transmit signals to the actin cytoskeleton. This lipid influences the activity of several actin-associated proteins that regulate the architecture of actin cytoskeleton. Signaling intermediates include focal adhesion molecules, such as vinculin and members of ERM and WASP [27]. Willebrand factor (vWF), ADAMTS13 and S100 calcium binding protein A7; Protein S100-A7 (S100A7) are key proteins that regulate blood hemostasis. Their altered concentration may contribute to endothelial dysfunction. Together with other clinical parameters, these proteins have also been used as candidate biomarkers for the diagnosis of acute ischemic stroke [28]. The cytoskeletal structure and cell junction are solely responsible for EC barrier integrity [29]. If the EC barrier is weakened, it should result in increased vascular permeability. Vinculin is an actin-binding protein. In the present study, the interaction of F-actin with vinculin was found to be uniformly distributed in the mock control cells. However, the vinculin pattern in the treated cells contrasted with that in the control cells, in which the protein pattern was a dot-like structure. Vinculin is also a component of cadherin- and integrin-based adhesion complexes, which play a role in maintaining cell integrity [30,31]. Vimentin has been reported to be involved in replication of DENV by interacting with NS4A in the replication complexes of DENV [32]. The involvement of moesin phosphorylation has been suggested as a candidate factor in the alteration of the F-actin cytoskeleton and the increase in EC monolayer permeability by activation of the p38 MAPK signaling pathway [33]. Recent studies showed that DENV NS1 from all four serotypes and other flaviviruses, including Zika and West Nile, can induce endothelial hyperpermeability in human EC lines, as measured by TEER [34,35]. Moreover, the interaction of NS1 with vascular endothelium results in the disruption of endothelial glycocalyx during a DENV infection [36,37]. Another possibility is that transient disruption of intercellular junctions, along with glycocalyx, may be responsible for vascular leakage [38]. A more specific mechanism of dengue NS1 has been described as the activation of p38 MAPK pathway, which results in a decrease in barrier integrity in primary human ECs [39].

The present work reports that the important roles of cytoskeleton proteins become imbalanced due to DENV infection and TNF-α induction. This phenomenon has been proposed as one of the major factors contributing to vascular leakage in the clinical manifestation of DHF/DSS pathogenesis. Knowledge of the cytoskeleton protein functions provides a better understanding of the synergistic effects and the roles of cytoskeleton proteins.

A low expression of moesin, alterations to actin stress fibers, and a dot-like structural formation of vinculin are responsible for the cellular permeability changes of human ECs during dengue virus infection and TNF-α induction.

Endothelial dysfunction leading to increased vascular permeability is a hallmark of severe dengue, leading to leakage of fluid into the pleural and peritoneal cavities, and shock. TNF-α is highly elevated in dengue patient, and it is likely to result in increased vascular permeability. The roles of the DENV-NS1 antigen and lipid mediators such as platelet activating factor (PAF) in causing vascular leakage have also been described [40]. The antigen and mediators raise the possibility of using drugs to block PAF receptors. In addition, the downstream mediator pathways generated by PAF may prove to be helpful in the treatment of severe disease. An understanding of the mechanisms of the several pathways that co-contribute to vascular leakage will lead to intervention in the pathogenesis. The use of antibodies against DENV-NS1 to reduce disease pathogenesis and provide protection against a lethal dengue challenge recently emerged as a candidate idea. This is because DENV-NS1 was found to reduce viral load, secreted-NS1 levels, and vascular leakage in a mouse model [35,41]. Moreover, it is important to establish whether other mediators play a role in the pathogenesis of DENV infections, and to determine if those mediators have the potential to be developed as therapeutics for the treatment of the infections.

## 5. Conclusions

Vascular leakage, one of the severe pathogeneses in DENV infections, was increased by DENV2 infection and TNF-α treatment in human endothelial (EA.hy926) cells. Analysis of cytoskeleton proteins in EA.hy926 cells treated by DENV2 and TNF-α revealed differential protein expressions. Following treatment with DENV2 and TNF-α, transendothelial permeability in EA.hy926 cells was associated with a decrease in moesin expression and with the presence of actin stress fibers and a dot-like pattern of vinculin. These effects lead to vascular leakage in DENV patients. Overexpression of moesin in human ECs treated with DENV and TNF-α could restore the function of transendothelial permeability. Improving our knowledge of the role of cytoskeleton proteins in maintaining membrane structure and integrity will enhance our understanding of the mechanism of vascular leakage in dengue patients with DHF and DSS.

## Figures and Tables

**Table 1:** Classification of molecular function of altered proteins in DENV2 plus TNF-α-treated EA.hy926 cells using PANTHER version 11.1 (Protein ANalysisTHrough Evolutionary Relationships).

Molecular Function	Amount %
structural molecule activity	23.10
transporter activity	4.40
receptor activity	3.10
catalytic activity	24.40
antioxidant activity	1.0
signal transducer activity	0.3
translation regulator activity	0.7
nucleic acid binding, enzyme modulator proteins and others	43

## Data Availability

Not applicable.

## Supplementary Materials

The following are available online at https://www.mdpi.com/article/10.3390/v13102042/s1, Supporting Information S1. Infection rate of dengue virus (DENV) infection in human ECs (EA.hy926) during mock, DENV2, TNF-α, and TNF-α plus DENV2 at various post-infection timepoints (A). The percentage of cell death in EA.hy926 after treatment with mock, DENV2, TNF-α, and TNF-α plus DENV2 at various post-infection timepoints (B). Supporting Information S2. Western blot analysis of cytoskeleton protein preparation from EA.hy926 cells. Antibodies against GAPDH and vimentin were used as markers for cytosolic and cytoskeleton proteins fractions, respectively. Supporting Information S3. Summary of altered proteins analyzed from EA.hy926 cells during treatment with TNF-α plus DENV2, using mass spectrometry and a MASCOT search engine of the database. Supporting Information S4. Summary of altered structural and cytoskeleton proteins from EA.hy926 cells during treatment with TNF-α plus DENV2.
