# Supplementary material for: Altered Moesin and Actin Cytoskeleton Protein Rearrangements Affect Transendothelial Permeability in Human Endothelial Cells upon Dengue Virus Infection and TNF-α Treatment
Source: Viruses. 2021 Oct 11;13(10):2042. doi: 10.3390/v13102042 (PMC8537470; doi:10.3390/v13102042)
Supplement: Supplementary file 1 [file viruses-13-02042-s001.zip › viruses-1368547-supplementary.pdf]

Supplement 1

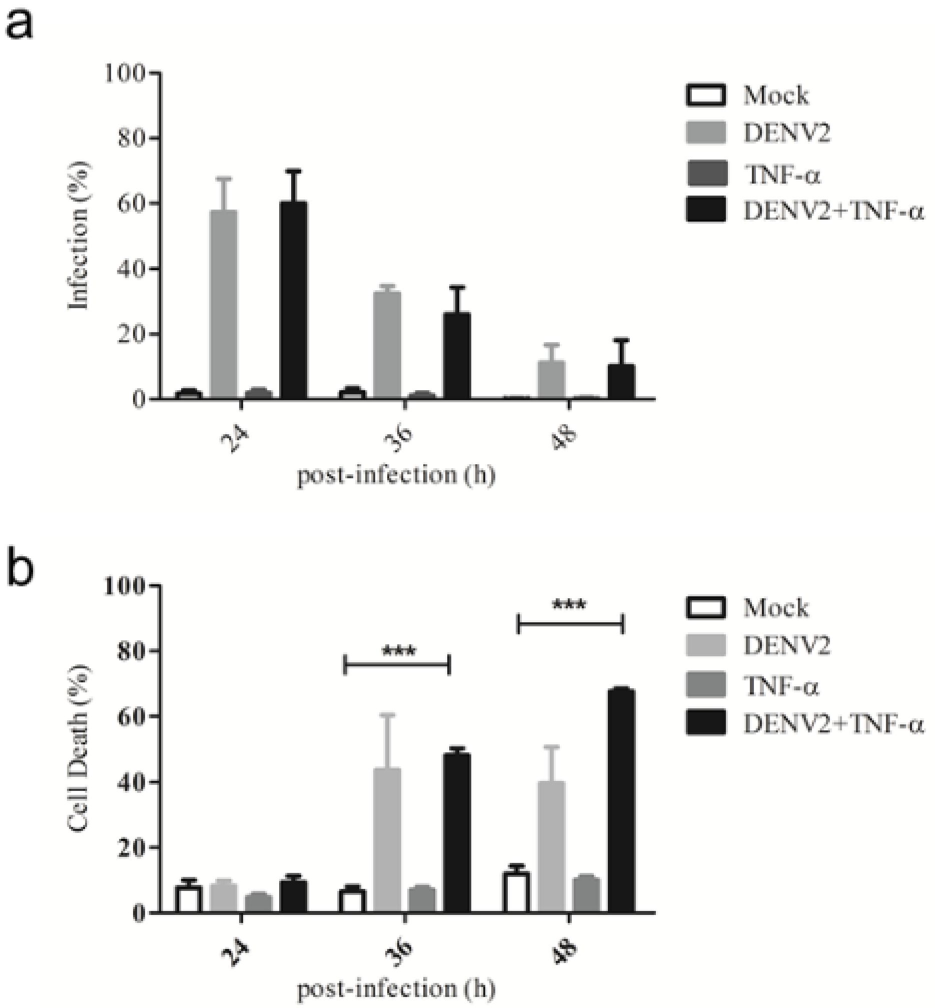

Supplement 2

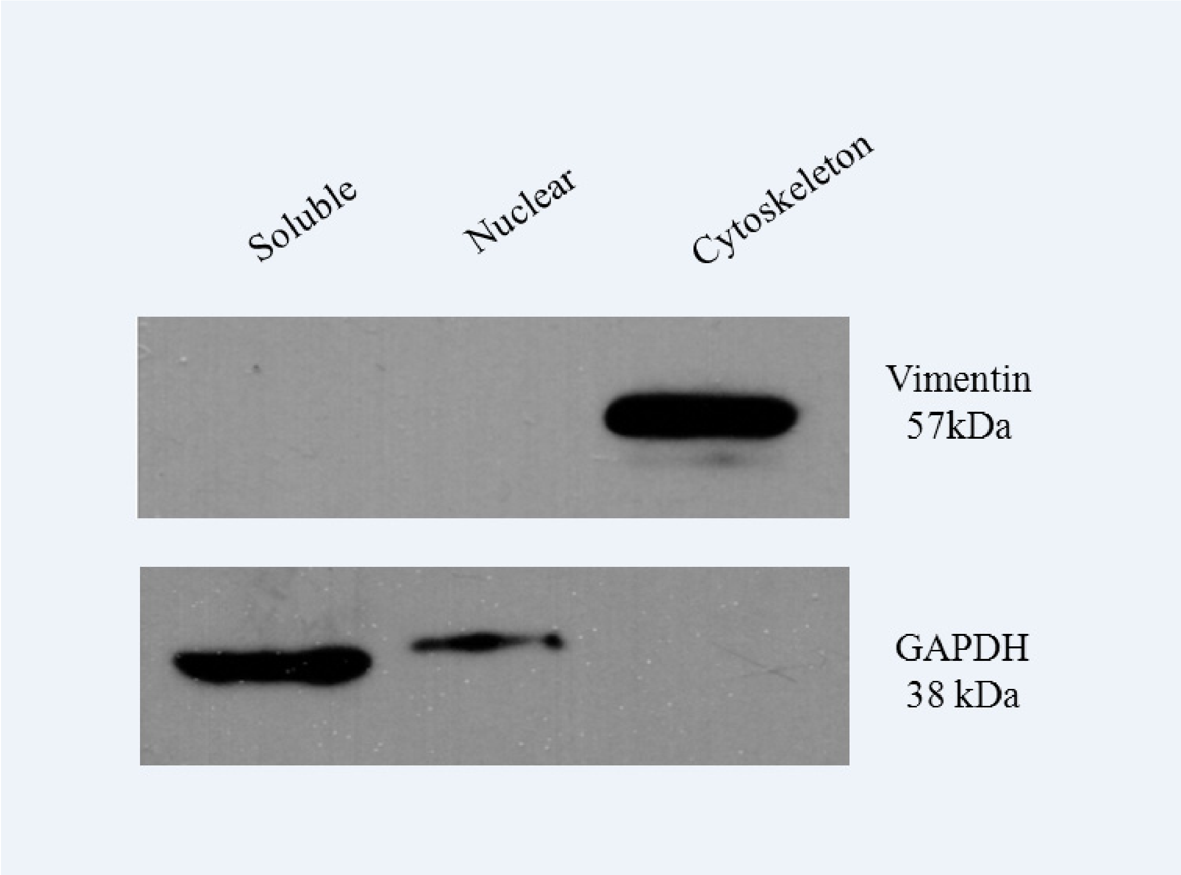

Supplement 3. Altered cytoskeleton proteins in EA.hy926 cells during DENV2 infection and TNF-α treatment.

No.	NCBI ID	Protein	pI	MW (kDa)	Identification score	No. of matched peptides	%cov	Mock (Mean±SD)	Treated (Mean±SD)	Ratio (Treated/Mock)
1	gi|296164	156 kDa Protein	5.52	156	73	2	6	0±0	no emPAI	n/a
2	gi|4506707	40S ribosomal protein S25	10.12	13.73	73	3	8	0.25±0	0±0	n/a
3	gi|407308	54 kDa protein	8.66	54.22	71	2	8.9	0.03±0.03	no emPAI	n/a
4	gi|31542947	60 kDa heat shock protein, mitochondrial	5.7	61.02	1001	17	45.9	0.96±0.69	0.72±0.68	0.75
5	gi|4506631	60S ribosomal protein L30	9.65	12.78	95	2	36.5	0.44±0.17	0±0	0.00
6	gi|306891	90kDa heat shock protein	4.97	83.24	254	8	9.3	0.11±0.08	0.06±0.06	0.53
7	gi|4501881	actin, alpha skeletal muscle	5.23	42.02	705	15	41.4	1.84±0.38	no emPAI	n/a
8	gi|4501887	actin, cytoplasmic 2	5.31	41.77	1170	17	61.6	4.02±0.78	2.65±1.10	0.66
9	gi|212293358	actinin alpha 1 isoform 3	5.38	107.1	157	4	5.1	0±0	0.03±0.02	n/a
10	gi|62421162	actin-like protein	6.19	11.53	86	3	28.2	0.3±0	0.03±0.02	0.08
11	gi|62420965	actin-like protein	6.04	11.51	92	3	19.4	0.3±0	0.13±0.12	0.42
12	gi|62421170	actin-like protein	6.06	11.55	115	3	24.3	0.94±0.25	0.51±0.19	0.54
13	gi|62421077	actin-like protein	6.19	11.6	62	2	10.7	0.3±0	0.25±0.24	0.83
14	gi|339920	ADP/ADT translocator protein	9.84	33.13	257	8	30	0.47±0	0.39±0.37	0.83
15	gi|179247	ADP/ATP carrier protein	9.71	32.96	308	9	36.6	0.67±0	0.27±0.25	0.39
16	gi|46852164	alpha-mannosidase 2C1 isoform 1	6.1	115.8	65	2	7.7	no emPAI	0.04±0.04	n/a
17	gi|444738085	alternative protein APLP2	12.06	18.99	61	2	19.9	no emPAI	0±0	n/a
18	gi|444738203	alternative protein HRC	11.99	34.32	60	2	35.1	no emPAI	0.46±0.46	n/a
19	gi|119590685	amyotrophic lateral sclerosis 2 (juvenile) chromosome region, candidate 11, isoform CRA_c, partial	7.34	208.9	61	2	3.2	0±0	0.83±0.42	n/a
20	gi|34391502	anion exchanger SLC4A3	6.01	138.6	79	3	5.7	no emPAI	0±0	n/a
21	gi|119626693	ankyrin 2, neuronal, isoform CRA_b	5.02	431.1	108	3	3.3	no emPAI	0.01±0.01	n/a
22	gi|739514	ankyrin B:ISOTYPE=440kD	5.03	430.1	86	2	1.9	0±0	no emPAI	n/a
23	gi|608025	ankyrin G	6.12	480.1	68	2	1.2	no emPAI	0±0	n/a

No.	NCBI ID	Protein	pI	MW (kDa)	Identification score	No. of matched peptides	%cov	Mock (Mean±SD)	Treated (Mean±SD)	Ratio (Treated/Mock)
24	gi|16306978	Annexin A2	7.57	38.59	1243	17	74.9	6.21±0.48	no emPAI	n/a
25	gi|18645167	Annexin A2	7.57	38.55	1237	17	78.5	6.25±0.49	no emPAI	n/a
26	gi|119597993	annexin A2, isoform CRA_c	5.93	32.43	1132	17	82.2	3.38±3.38	0±0	0.00
27	gi|415819	antigen of the monoclonal antibody Ki-67	9.45	358.5	111	3	4.1	no emPAI	0±0	n/a
28	gi|182397	APC	7.84	311.5	69	2	3.5	no emPAI	0.03±0.03	n/a
29	gi|16118245	ARAP2	7.01	193.3	66	2	2.8	no emPAI	0±0	n/a
30	gi|56417899	ARF-binding protein 1	5.1	481.6	66	2	2	no emPAI	0±0	n/a
31	gi|4502247	armadillo repeat protein deleted in velo-cardio-facial syndrome	6.38	104.6	99	3	8.5	no emPAI	0±0	v
32	gi|5453559	ATP synthase subunit d, mitochondrial isoform a	5.21	18.48	64	2	26.1	0.18±0	0±0	0.00
33	gi|4502303	ATP synthase subunit O, mitochondrial precursor	9.97	23.26	198	5	29.1	0.79±0.12	no emPAI	n/a
34	gi|16877071	ATP synthase, H+ transporting, mitochondrial F1 complex, gamma polypeptide 1	9.23	32.95	124	4	8.4	0.33±0	0±0	0.00
35	gi|119630463	ATP-binding cassette, sub-family C (CFTR/MRP), member 13, isoform CRA_a	9.13	107.1	65	2	4.5	no emPAI	0.24±0.24	n/a
36	gi|28935	ATP-citrate (pro-S-)-lyase	6.42	121.3	69	2	4	no emPAI	0.08±0.08	n/a
37	gi|100913206	ATP-dependent RNA helicase A	6.41	140.9	133	3	9.8	0.02±0	0.03±0.02	1.25
38	gi|825671	B23 nucleophosmin (280 AA)	4.71	30.92	340	9	26.1	1.06±0.31	0.42±0.23	0.39
39	gi|86947	beta-galactoside-binding lectin, placental - human (fragments)	4.63	11.99	112	3	32.7	0.29±0	0±0	0.00
40	gi|12053672	beta-myosin heavy chain	5.63	223	72	2	6.1	0±0	0.11±0.06	n/a
41	gi|4507339	brachyury protein isoform 1	6.62	47.41	61	2	6.2	0.04±0.04	0.09±0.09	2.57
42	gi|6683494	bromodomain adjacent to zinc finger domain 1A	7.76	190.7	82	3	4.1	0.02±0	0.04±0.03	1.75
43	gi|306875	C protein	5.1	31.95	218	6	20	0.43±0.30	0.21±0.14	0.48

No.	NCBI ID	Protein	pI	MW (kDa)	Identification score	No. of matched peptides	%cov	Mock (Mean±SD)	Treated (Mean±SD)	Ratio (Treated/Mock)
44	gi|119603980	Ca2+-dependent activator protein for secretion 2, isoform CRA_d	5.8	147.8	69	2	6.8	no emPAI	0.85±0.85	n/a
45	gi|189571674	cadherin-23 isoform 1 precursor	4.5	369.3	60	2	1.6	no emPAI	0.06±0.06	n/a
46	gi|119585699	calcium channel, voltage-dependent, L type, alpha 1D subunit, isoform CRA_j	6.96	242.9	74	3	3.9	0±0	no emPAI	n/a
47	gi|21541504	calcium-dependent activator protein for secretion protein	5.53	144	86	3	6.4	no emPAI	0±0	n/a
48	gi|10716563	calnexin precursor	4.47	67.53	94	3	10.1	0±0	no emPAI	n/a
49	gi|180631	calnexin, partial	4.22	40.81	76	2	11.2	0.08±0	0.01±0.01	0.13
50	gi|17298317	candidate tumor suppressor protein, partial	4.78	172.4	64	2	5.7	0±0	0.02±0.01	n/a
51	gi|219524	cardiac alpha-myosin heavy chain	5.63	223.4	114	3	4.5	0.01±0	no emPAI	n/a
52	gi|29727	cardiac beta myosin heavy chain	5.62	222.8	79	3	5.9	0±0	no emPAI	n/a
53	gi|951338	CAS	5.51	110.2	65	2	4	0.03±0	no emPAI	n/a
54	gi|10835165	CD59 glycoprotein preproprotein	6.02	14.17	95	3	15.6	0.24±0	0±0	0.00
55	gi|50083279	centrosomal protein of 135 kDa	5.87	133.4	61	2	7.3	no emPAI	0±0	n/a
56	gi|109255230	centrosomal protein of 170 kDa isoform beta	6.77	164.4	73	2	6.2	no emPAI	0±0	n/a
57	gi|50083293	centrosomal protein of 89 kDa	6.36	89.53	100	2	10.1	0±0	no emPAI	n/a
58	gi|4680717	CGI-39 protein	6.92	15.73	60	2	20	0.22±0	0±0	0.00
59	gi|11527207	CGI-85	9.29	30.31	63	2	23	no emPAI	0±0	n/a
60	gi|145580001	Chain A, Crystal Structure Of A Fragment Of The Plakin Domain Of Plectin	7.18	42.13	250	7	24.3	0.12±0	no emPAI	n/a
61	gi|395759492	Chain A, Crystal Structure Of A Heat Shock 70kda Protein 2 (Hspa2) From Homo Sapiens At 1.80 A Resolution	7.18	42.13	250	7	24.3	0.28±0.05	no emPAI	n/a
62	gi|349587814	Chain A, Crystal Structure Of Full-Length Human Peroxiredoxin 4 In The Reduced Form	6.1	27.95	67	2	13	0.25±0	0.14±0.01	0.54

No.	NCBI ID	Protein	pI	MW (kDa)	Identification score	No. of matched peptides	%cov	Mock (Mean±SD)	Treated (Mean±SD)	Ratio (Treated/Mock)
63	gi|83753119	Chain A, Crystal Structure Of Human Full-Length Vinculin (Residues 1-1066)	5.34	14.73	157	4	60	no emPAI	0.06±0.06	n/a
64	gi|365813094	Chain A, Crystal Structure Of Human Galectin-1 In Complex With Methyl 2-o- Acetyl-3-o-toluoyl-beta-d-talopyranoside	5.34	14.73	157	4	60	0.26±0.26	no emPAI	n/a
65	gi|358440049	Chain A, Crystal Structure Of Human Galectin-7 In Complex With A Galactose-Benzylphosphate Inhibitor	7.18	14.73	85	3	21.8	0.23±0	0.15±0.15	0.67
66	gi|609412460	Chain A, Crystal Structure Of Human Lnkh2b-h2a.z-anp32e	10.22	22.46	310	9	12.9	2.35±0.21	1.23±0.67	0.52
67	gi|347447296	Chain A, Crystal Structure Of Human Nucleosome Core Particle Containing H3k79q Mutation	11.12	15.68	80	3	12.9	0.22±0	0±0	0.00
68	gi|110590538	Chain A, Crystal Structure Of Human Pyrroline-5-carboxylate Synthetase	5.97	51.1	107	3	19	0±0	no emPAI	n/a
69	gi|122920033	Chain A, Crystal Structure Of Rhogef Protein Asef	5.15	54.91	108	3	16.4	no emPAI	0.50±0.50	n/a
70	gi|374414590	Chain A, Crystal Structure Of StabilisedVimentin Coil2 Fragment	5.41	8.667	307	9	79.2	2.52±0.53	no emPAI	n/a
71	gi|520729524	Chain A, Crystal Structure Of Substrate-free Human Presequence Protease	5.09	114.1	78	3	9.5	no emPAI	0.10±0.10	n/a
72	gi|134105326	Chain A, Crystal Structure Of The First Hin-200 Domain Of Interferon-Inducible Protein 16	9.39	23.63	62	2	16.5	no emPAI	0.03±0.03	n/a
73	gi|61680421	Chain A, Crystal Structure Of The Hspbp1 Core Domain	5.92	32.79	76	2	23.6	no emPAI	0±0	n/a
74	gi|290560068	Chain A, Crystal Structure Of The Humanized Recombinant Fab Fragment Of A Murine; Antibody	9.42	24.86	62	2	19.2	0.13±0	0±0	0.00
75	gi|342350777	Chain A, Human Annexin V With Incorporated Methionine Analogue Azidohomoalanine	4.89	35.75	225	10	29.1	0.47±0.06	0.35±0.30	0.74

No.	NCBI ID	Protein	pI	MW (kDa)	Identification score	No. of matched peptides	%cov	Mock (Mean±SD)	Treated (Mean±SD)	Ratio (Treated/Mock)
76	gi|157833780	Chain A, Human Annexin V With Proline Substitution By Thioproline	4.94	35.98	248	11	32.2	0.46±0.06	no emPAI	n/a
77	gi|325533397	Chain A, Human Lamin A Coil 2b Fragment	5.46	8.661	231	7	60.8	1.69±1.02	0.03±0.03	0.02
78	gi|20149844	Chain A, Human Vimentin Coil 1a Fragment (1a)	4.6	4.646	188	7	76.9	9.3±0	no emPAI	n/a
79	gi|723586843	Chain A, Mitochondrial Adck3 Employs An Atypical Protein Kinase-like Fold To Enable Coenzyme Q Biosynthes	5.93	44.86	86	3	14.2	no emPAI	0.19±0.10	n/a
80	gi|392935428	Chain A, Nmr Structure Of Npm1_c70	9.52	8.494	130	3	47.3	0±0	no emPAI	n/a
81	gi|159162145	Chain A, Rotamer Strain As A Determinant Of Protein Structural Specificity	6.56	8.56	95	3	61.8	0.6±0.27	0.26±0.17	0.42
82	gi|88191927	Chain A, Structural Basis For Recognition Of Uuuoh 3~-Terminii Of Nascent Rna Pol Iii Transcripts By La Autoantigen	5.89	22.66	71	2	19	0±0	0.08±0.08	n/a
83	gi|47169316	Chain A, Structure Of Pitp-Alpha Complexed To Phosphatidylinositol	5.98	31.63	61	2	7.8	0±0	no emPAI	n/a
84	gi|31615803	Chain A, Synthetic Ubiquitin With Fluoro-Leu At 50 And 67	6.56	8.555	128	3	77.6	1.26±0.38	0±0.47	0.00
85	gi|809185	Chain A, The Effect Of Metal Binding On The Structure Of	4.94	35.78	287	9	31.7	0.47±0.06	0.01±0.01	0.02

No.	NCBI ID	Protein	pI	MW (kDa)	Identification score	No. of matched peptides	%cov	Mock (Mean±SD)	Treated (Mean±SD)	Ratio (Treated/Mock)
		Annexin V And Implications For Membrane Binding								
86	gi|9955007	Chain A, Thioredoxin Peroxidase B From Red Blood Cells	5.44	21.8	78	3	13.2	0.15±0	no emPAI	n/a
87	gi|56554343	Chain A, X-Ray Crystal Structure Of C2s Human Galectin-1 Complexed With Galactose	5.12	14.63	159	4	50	0.52±0	0.23±0.12	0.44
88	gi|229597861	Chain A, X-Ray Crystal Structure Of Coil 1a Of Human Vimentin	4.35	4.674	198	5	76.9	4.1±3.72	5.24±2.85	1.28
89	gi|42542977	Chain A, X-Ray Crystal Structure Of Human Galectin-1	5.34	14.58	123	5	57.5	0.26±0.26	no emPAI	n/a

No.	NCBI ID	Protein	pI	MW (kDa)	Identification score	No. of matched peptides	%cov	Mock (Mean±SD)	Treated (Mean±SD)	Ratio (Treated/Mock)
90	gi|290790186	Chain A, X-ray Crystal Structure Of Trf1 And Fbx4 Complex	5.44	24.36	84	3	16.7	no emPAI	0.35±0.33	n/a
91	gi|374074124	Chain B, Crystal Structure Of Human N12-I15, An Adcc And Non-Neutralizing Anti- Hiv-1 Env Antibody	6.48	23.81	61	2	15.1	0.14±0	0±0	0.00
92	gi|347447327	Chain B, Crystal Structure Of Human Nucleosome Core Particle Containing H4k31q Mutation	11.36	11.64	171	4	41.5	1.17±0	0.77±0.33	0.60
93	gi|635576697	Chain B, Crystal Structure Of SchizosaccharomycesPombe Sst2 Catalytic Domain And Ubiquitin	6.78	8.971	94	3	63	0.31±0.44	no emPAI	n/a
94	gi|520729525	Chain B, Crystal Structure Of Substrate-free Human Presequence Protease	5.02	114	64	2	10.5	no emPAI	0±0	n/a
95	gi|428697911	Chain B, Structure Of Bcl-xl Bound To Bimlock	4.01	2.408	63	2	100	no emPAI	0±0	n/a
96	gi|208435632	Chain C, Structural Insights Into Nedd8 Activation Of Cullin-Ring Ligases: Conformational Control Of Conjugation	8.97	44.63	115	3	18.1	no emPAI	0±0	n/a
97	gi|695722159	Chain D, Structure Of Bg505 Sosip.664 In Complex With Broadly Neutralizing Antibodies, Pgt122 And 35o22	9.04	26.13	61	2	8.6	0.13±0	0±0	0.00
98	gi|340780331	Chain D, The Structure Of The Centromeric Nucleosome Containing Cenp-A	10.31	14.12	187	5	41.9	0.46±0.65	no emPAI	n/a
99	gi|530537677	Chain E, Structure Of An Rsp5xubxsna3 Complex: Mechanism Of Ubiquitin Ligation And Lysine Prioritization By A Hect E3	7.06	9.559	127	4	73.5	0.51±0.73	0.43±0.35	0.84
100	gi|588293128	Chain H, Crystal Structure Of Human Fab Cap256-vrc26.10, A Potent V1v2-directed Hiv-1 Neutralizing Antibody	8.44	27.61	64	2	23.8	0.12±0	0±0	0.00
101	gi|230867	Chain R, Twinning In Crystals Of Human Skeletal Muscle D-	6.6	35.85	209	4	15.6	0.14±0.2	0.05±0.04	0.32

No.	NCBI ID	Protein	pI	MW (kDa)	Identification score	No. of matched peptides	%cov	Mock (Mean±SD)	Treated (Mean±SD)	Ratio (Treated/Mock)
		Glyceraldehyde-3- Phosphate Dehydrogenase								
102	gi|13562153	channel-kinase 1	8.16	212.5	65	2	4.1	0.08±0.06	0±0	0.00
103	gi|306890	chaperonin (HSP60)	5.7	60.99	954	17	46.2	1.28±0.11	0.04±0.03	0.03
104	gi|3169116	cICK0721Q.2 (60S Ribosomal Protein L12 LIKE protein)	9.57	17.92	112	3	13.3	0.19±0	0±0	0.00
105	gi|359385708	cilia- and flagella-associated protein 46	7.07	303.3	68	2	3.6	0±0	no emPAI	n/a
106	gi|4758012	clathrin heavy chain 1 isoform 1	5.48	191.5	114	3	5.3	0.02±0	0.27±0.38	11.43
107	gi|182383	coagulation factor VIII	6.95	266.8	87	3	5.3	0.01±0	0±0	0.00
108	gi|5031635	cofilin-1	8.22	18.49	105	3	25.9	0.27±0.19	0±0.08	0.00
109	gi|38348729	coiled-coil domain-containing protein 171	6.37	152.7	98	3	7.9	no emPAI	0±0	n/a

No.	NCBI ID	Protein	pI	MW (kDa)	Identification score	No. of matched peptides	%cov	Mock (Mean±SD)	Treated (Mean±SD)	Ratio (Treated/Mock)
110	gi|32140760	collagen alpha-1(XXVII) chain preproprotein	9.83	186.8	86	3	8.9	no emPAI	0±0	n/a
111	gi|116256354	collagen alpha-2(IV) chain preproprotein	8.89	167.4	64	2	4.6	0±0	0.68±0.32	n/a
112	gi|156616290	collagen alpha-6(VI) chain precursor	6.44	247	60	2	5.3	0±0	no emPAI	n/a
113	gi|13436464	CPSF6 protein	6	52.29	93	3	10.5	0.03±0.03	0±0	0.00
114	gi|181250	cyclophilin, partial	9.33	22.6	62	2	6.3	0.15±0	0±0	0.00
115	gi|381234392	cytochrome c oxidase subunit II (mitochondrion)	4.67	25.55	118	3	11.9	0.28±0	0.07±0.07	0.23
116	gi|545768875	cytochrome c oxidase subunit II (mitochondrion)	4.67	25.6	67	2	16.3	0±0	0.07±0.06	n/a
117	gi|310776861	cytochrome c oxidase subunit II	4.76	25.54	117	3	14.5	0.28±0	0±0	0.00
118	gi|310777477	cytochrome c oxidase subunit II	4.74	25.56	117	3	11.9	0.28±0	0.07±0.07	0.23
119	gi|545768329	cytochrome c oxidase subunit II, partial (mitochondrion)	4.59	25.07	152	4	12.2	0.28±0	0.07±0.06	0.23
120	gi|34035	cytokeratin 18 (232 AA)	4.92	26.43	302	9	39.2	0.71±0.1	0.10±0.10	0.13
121	gi|30311	cytokeratin 18 (424 AA)	5.27	47.31	1072	17	56.6	1.9±0.40	0.88±0.73	0.46
122	gi|181390	cytokeratin 2	8.38	65.83	89	4	5	0±0	0.03±0.02	n/a
**No.**	**NCBI ID**	**Protein**	**pI**	**MW (kDa)**	**Identification score**	**No. of matched peptides**	**%cov**	**Mock (Mean±SD)**	**Treated (Mean±SD)**	**Ratio (Treated/Mock)**
123	gi|181573	cytokeratin 8	5.52	53.53	877	11	30.7	1.18±0.21	no emPAI	n/a
124	gi|435476	cytokeratin 9	5.19	62.09	437	11	25.4	0.59±0	0±0	0.00
125	gi|3901030	cytokeratin type II	7.6	59.47	266	8	10.5	0.26±0.03	0±0	0.00
126	gi|2580550	dead box, X isoform	6.73	73.23	96	3	8.3	0±0	0.01±0.01	n/a
127	gi|119581895	dedicator of cytokinesis 2, isoform CRA_a	6.68	215.8	94	3	4.7	0±0	1.44±0.68	n/a
128	gi|51095134	dedicator of cytokinesis 4	7.44	225.1	64	2	3	no emPAI	0±0	n/a
129	gi|40804411	deubiquitinating enzyme 3	8.05	59.57	84	3	10.9	no emPAI	0.06±0.06	n/a
130	gi|2181867	DFFRY	6.84	95.63	86	3	8.2	no emPAI	0±0	n/a
131	gi|62088586	diacylglycerol kinase, theta variant	5.06	240.9	67	2	2.8	no emPAI	0±0	n/a
132	gi|181575	dihydrolipoamide dehydrogenase precursor	8.2	54.24	77	3	7.5	0±0	1.78±2.44	n/a
133	gi|4506675	dolichyl-diphosphooligosaccharide--protein glycosyltransferase subunit 1 precursor	5.96	68.53	102	3	6.6	0.07±0.02	0.01±0.01	0.15
134	gi|209413738	dolichyl-diphosphooligosaccharide--protein glycosyltransferase subunit 2 isoform 2 precursor	5.7	67.68	62	2	3.3	0.05±0	0±0	0.00
135	gi|7963632	dual oxidase	8.12	177.2	90	4	5.3	no emPAI	0±0	n/a
136	gi|222144249	dynein heavy chain domain-containing protein 1 isoform 1	6.25	533.3	104	3	2.6	no emPAI	0±0	n/a
137	gi|119585619	dynein, axonemal, heavy polypeptide 1, isoform CRA_a	5.7	499.1	64	2	3.1	0±0	0.39±0.39	n/a
138	gi|119602169	dynein, cytoplasmic 1, heavy chain 1, isoform CRA_e	6.01	403.1	80	3	3.6	no emPAI	0±0	n/a
139	gi|663429604	E3 ubiquitin-protein ligase TRIM36 isoform 4	5.58	65.53	78	3	12	no emPAI	0±0	n/a
140	gi|704416	elongation factor Tu	7.7	49.51	73	3	6.6	0.07±0	0±0	0.00
141	gi|7705029	EMT	7.51	71.87	82	3	6.3	no emPAI	0±0	n/a
142	gi|307133775	endoribonuclease Dicer isoform 2	5.5	208.3	91	4	7.9	no emPAI	0±0	n/a
**No.**	**NCBI ID**	**Protein**	**pI**	**MW (kDa)**	**Identification score**	**No. of matched peptides**	**%cov**	**Mock (Mean±SD)**	**Treated (Mean±SD)**	**Ratio (Treated/Mock)**
143	gi|526253086	endothelial differentiation-related factor 1 isoform 3	9.88	15.63	65	2	19.9	0±0	0.01±0.01	n/a
144	gi|862457	enoyl-CoA hydratase/3-hydroxyacyl-CoA dehydrogenase alpha-subunit of trifunctional protein	6.56	231.5	67	2	3	0.04±0	4.10±3.87	n/a
145	gi|181402	epidermal cytokeratin 2	8.07	65.83	336	9	9.1	0.41±0	4.75±4.41	11.51
146	gi|14250470	ERO1-like (S. cerevisiae)	5.62	54.36	89	4	12	no emPAI	0±0	n/a
147	gi|927065	eukaryotic translation elongation factor 1 alpha 1-like 14	8.54	42.8	105	3	13.6	0.12±0.04	0±0	0.00
148	gi|63020636	expressed in synovial lining protein	9.23	199.3	80	2	4.4	no emPAI	0.25±0.25	n/a
149	gi|6063147	ezrin	9.19	19.22	84	3	23.9	0.18±0	0±0	0.00
150	gi|4502477	F-box/WD repeat-containing protein 1A isoform 2	8.24	65.01	68	2	4.4	0±0	0.03±0.02	n/a
151	gi|46559358	fibrillin 1	4.81	312	101	3	9.5	no emPAI	0±0	n/a

152	gi|119593150	filamin A, alpha (actin binding protein 280), isoform CRA_a	5.7	248.4	117	3	5.9	no emPAI	0±0	n/a
153	gi|744518	FKBP-rapamycin-associated protein	6.81	288.8	102	3	8.6	no emPAI	0.33±0.33	n/a
154	gi|260268505	FLJ00343 protein	5.77	281.3	145	4	7.3	no emPAI	0±0	n/a
155	gi|119631344	FLJ44048 protein, isoform CRA_a	6.11	681.9	83	3	1.8	no emPAI	0±0	n/a
156	gi|33341656	FP1047	6.76	69.35	140	4	14.1	0.07±0.02	0±0	0.00
157	gi|58531795	G protein-coupled receptor 112	5.83	333.2	85	3	2.1	no emPAI	0.18±0.16	n/a
158	gi|31645	glyceraldehyde-3-phosphate dehydrogenase	8.26	36.03	317	9	31.3	0.55±0	0.21±0.20	0.38
159	gi|119567961	hCG1643231, isoform CRA_b, partial	8.21	35.28	75	2	7.7	0±0	0.05±0.05	n/a
160	gi|119585743	hCG1779312, isoform CRA_a	5.63	452.5	139	4	5.1	no emPAI	0±0	n/a
161	gi|119598268	hCG1786960, partial	5.04	21.66	140	4	14.6	0.33±0	0±0	0.00
162	gi|119607348	hCG1810953, isoform CRA_c	6.9	89.19	96	3	13.9	no emPAI	0±0	n/a
163	gi|119619489	hCG1811351	8.71	102.9	88	4	5.4	no emPAI	0±0	n/a
164	gi|119620733	hCG1818387	6.3	14.49	66	2	30.3	0±0	0.12±0.11	n/a

No.	NCBI ID	Protein	pI	MW (kDa)	Identification score	No. of matched peptides	%cov	Mock (Mean±SD)	Treated (Mean±SD)	Ratio (Treated/Mock)
165	gi|119607256	hCG1988300, isoform CRA_a	5.09	47.13	362	9	22.8	0.28±0.11	0.29±0.27	1.01
166	gi|119620881	hCG1988320, isoform CRA_b	4.65	36.12	95	3	11.7	0.09±0	0±0	0.00
167	gi|119573587	hCG1997137, isoform CRA_a	4.99	29.13	76	2	10	0±0	0.12±0.01	n/a
168	gi|119589777	hCG2005194, partial	9.4	25.24	63	2	15.3	no emPAI	0±0	n/a
169	gi|119626698	hCG2042471, partial	5.14	5.344	87	3	35.4	0.68±0	0±0	0.00
170	gi|119576791	hCG2043250, isoform CRA_b	9.23	111.2	74	3	4.6	0.02±0.02	0.23±0.12	15.33
171	gi|119620305	hCG21219, partial	6.19	106.8	180	5	6	0.03±0.03	0.02±0.02	0.50
172	gi|119631384	hCG22236, isoform CRA_b, partial	9.13	55.8	224	6	19.4	0.14±0.03	0.83±1.18	5.00
173	gi|119590144	hCG22803, isoform CRA_e	6.93	499.2	75	2	3.2	no emPAI	0±0	n/a
174	gi|55749742	HEAT repeat-containing protein 5B	6.77	224.2	73	3	4.6	no emPAI	0.06±0.06	n/a
175	gi|119590566	heat shock 10kDa protein 1 (chaperonin 10), isoform CRA_f	9.45	13.56	105	3	56.3	0.13±0.13	0±0	0.00
176	gi|12653415	Heat shock 70kDa protein 9 (mortalin)	6.03	73.68	171	4	8.2	0±0	0.08±0.06	n/a
177	gi|5729877	heat shock cognate 71 kDa protein isoform 1	5.37	70.85	503	11	25.9	0.38±0.09	0.29±0.23	0.76
178	gi|386785	heat shock protein	5.42	69.83	124	4	11.3	0.1±0	0.03±0.02	0.25
179	gi|4204880	heat shock protein	5.56	69.95	305	9	17.7	0.22±0.03	0.13±0.18	0.58
180	gi|639910	heat shock protein	7.85	13.76	86	3	38.4	0.25±0	0.31±0.44	1.25
181	gi|662841	heat shock protein 27	7.83	22.31	78	3	8	0.15±0	0±0	0.00
182	gi|188492	heat shock-induced protein	5.76	70.36	146	4	12.6	0.1±0	0.16±0.23	1.63
183	gi|14575679	hemicentin	6.05	613.3	97	3	4.4	no emPAI	0±0	n/a
184	gi|62898443	heterogeneous nuclear ribonucleoprotein H3 isoform a variant	6.8	36.9	98	3	14.2	0.09±0	2.38±2.24	26.39
185	gi|14043072	heterogeneous nuclear ribonucleoproteins A2/B1 isoform B1	8.97	37.41	436	11	51.6	0.97	0.41±0.34	0.42
186	gi|184484	hexabrachion	4.81	241.1	81	2	6.4	0±0	no emPAI	n/a
187	gi|50949256	hHa7 protein	4.93	45.74	92	4	7.3	0±0	0.24±0.18	n/a
188	gi|4504431	high mobility group protein HMGI-C	10.63	11.83	63	2	21.1	0.29±0	0.06±0.09	0.22

No.	NCBI ID	Protein	pI	MW (kDa)	Identification score	No. of matched peptides	%cov	Mock (Mean±SD)	Treated (Mean±SD)	Ratio (Treated/Mock)
		isoform a								
189	gi|119569461	Histidine acid phosphatase domain containing 1, isoform CRA_a	8.35	138	68	2	4.6	0±0	0.08±0.08	n/a
190	gi|6002696	histone acetyltransferase MORF beta	5.64	231.2	102	3	5.7	no emPAI	0.21±0.21	n/a
191	gi|4504239	histone H2A type 1	10.9	14.08	215	6	35.4	0±0	0.61±0.43	n/a
192	gi|1568551	histone H2B	10.31	13.93	277	8	52.4	5.52±0.71	1±1.41	0.18
193	gi|10800140	histone H2B type 1-B	10.31	13.94	279	8	52.4	0±0	1.81±1.81	n/a
194	gi|386772	histone H3, partial	10.99	15.24	71	2	15.6	0.07±0.1	4.76±4.33	64.91
195	gi|4504301	histone H4	11.36	11.36	65	2	18.4	0.61±0.61	0.23±0.12	0.38
196	gi|27544394	homologue to Drosophila photoreceptor protein calphotin	5.35	226.5	63	2	2.3	no emPAI	0±0	n/a
197	gi|321117267	HSPE1-MOB4 protein	5.79	29.72	94	3	22.6	0.11±0	0.06±0.05	0.50
198	gi|106322	hypothetical protein (L1H 3~ region) - human	9.64	149.3	64	2	3.1	0±0	0.39±0.20	n/a
199	gi|30722305	hypothetical protein	5.66	222.8	168	4	9.6	0.01±0	0±0	0.00

200	gi|5262477	hypothetical protein	5.35	77.1	62	2	11.4	no emPAI	0.18±0.18	n/a
201	gi|7018420	hypothetical protein	12.94	31.7	67	2	7.5	no emPAI	0±0	n/a
202	gi|12052750	hypothetical protein	5.35	22.2	63	2	30.6	no emPAI	0±0	n/a
203	gi|51476230	hypothetical protein	8.51	247.5	84	3	3	no emPAI	0±0	n/a
204	gi|51476571	hypothetical protein	5.24	122	61	2	8.4	no emPAI	1.34±1.34	n/a
205	gi|31874124	hypothetical protein	6.02	270.2	95	3	3.6	0±0	0.01±0.01	n/a
206	gi|57997573	hypothetical protein	4.71	27.16	74	2	25.4	0±0	0.06±0.06	n/a
207	gi|119571008	hypothetical protein DKFZp434N035, isoform CRA_b	8.47	47.51	62	2	12	no emPAI	0.05±0.05	n/a
208	gi|119568538	IBR domain containing 1, isoform CRA_e	9.14	35.18	62	2	14.7	no emPAI	0±0	n/a
209	gi|524453580	IgG H chain	9.54	25	66	2	9	0.13±0	no emPAI	n/a
210	gi|316996524	immunoglobulin gamma heavy chain	9.1	23.69	63	2	12.6	0.14±0	0±0	0.00
211	gi|39938102	immunoglobulin heavy chain variable region	9.2	16.1	67	2	29.5	0.21±0	0.01±0.01	0.05

No.	NCBI ID	Protein	pI	MW (kDa)	Identification score	No. of matched peptides	%cov	Mock (Mean±SD)	Treated (Mean±SD)	Ratio (Treated/Mock)
212	gi|39938240	immunoglobulin heavy chain variable region	9.13	15.9	67	2	14.2	0.21±0	no emPAI	n/a
213	gi|37696952	immunoglobulin heavy chain, VH region	8.6	13.65	63	2	21.9	0.25±0	0.09±0.09	0.36
214	gi|323432570	immunoglobulin variable region	8.51	15.46	73	3	22.7	0.22±0	0±0	0.00
215	gi|323432838	immunoglobulin variable region	8.83	14.41	61	2	38.5	0.24±0	0±0	0.00
216	gi|323432832	immunoglobulin variable region	9.41	15.77	63	2	30.6	0.22±0	0.03±0.03	0.14
217	gi|249616	insulin-like growth factor I receptor, IGF-IR [human, Peptide, 1367 aa]	5.58	154.7	61	2	3.7	no emPAI	0±0	n/a
218	gi|89213250	intermediate filament family orphan 1 isoform 4	4.89	62.44	68	2	9.6	no emPAI	0.14±0.14	n/a
219	gi|386848	keratin	5.09	51.63	86	3	4.9	0±0	0.03±0.03	n/a
220	gi|11935049	keratin 1	8.16	66.03	353	9	23.6	0.26±0.37	0.11±0.09	0.40
221	gi|375314771	keratin 1	8.15	66.01	720	11	32.9	0.93±0.05	0.53±0.53	0.57
222	gi|375314779	keratin 1	8.15	66.03	651	11	31.7	0.79±0.07	1.08±1.01	1.36
223	gi|119581085	keratin 10 (epidermolytic hyperkeratosis; keratosis palmaris et plantaris), isoform CRA_b	5.13	63.31	166	4	13.9	0±0	0.08±0.07	n/a
224	gi|21961605	Keratin 10	5.09	58.79	487	14	33.6	0.69±0.09	0±0	0.00
225	gi|12803709	Keratin 14	5.09	51.62	402	11	23.5	0.45±0	0.01±0.02	0.03
226	gi|31074631	keratin 1b	5.73	61.76	234	7	9.9	0.23±0	no emPAI	n/a
227	gi|20306864	Keratin 23 (histone deacetylase inducible)	6.09	48.15	66	2	2.1	0.31±0.33	0±0	0.00
228	gi|119617038	keratin 5 (epidermolysisbullosa simplex, Dowling-Meara/Kobner/Weber-Cockayne types), isoform CRA_a	5.91	38.55	367	9	28.9	0.69±0.07	0±0	0.00
229	gi|18999435	Keratin 5	7.59	62.34	393	9	19.2	0.38±0.03	0±0	0.00
230	gi|119617032	keratin 6B, isoform CRA_a	8.38	59.87	454	14	26.1	0.65±0.04	0±0	0.00
231	gi|119581148	keratin 9 (epidermolyticpalmoplantarkeratoderma)	6.95	57.53	414	11	26.7	0.56±0	0.03±0.03	0.05

No.	NCBI ID	Protein	pI	MW (kDa)	Identification score	No. of matched peptides	%cov	Mock (Mean±SD)	Treated (Mean±SD)	Ratio (Treated/Mock)
232	gi|186685	keratin type 16	4.97	50.67	78	3	6.4	0±0	0.04±0.04	n/a
233	gi|908803	keratin type II	8.09	60.19	476	14	27	0.64±0.04	0.02±0.03	0.03
234	gi|4504927	keratin, type I cuticular Ha6	4.9	52.21	91	4	6.2	0.03±0.03	0.06±0.03	2.00
235	gi|4557701	keratin, type I cytoskeletal 17	4.97	48.08	208	4	16.7	0.22±0	0.03±0.02	0.11
236	gi|4504935	keratin, type II cuticular Hb5 isoform 1	6.28	55.77	107	3	13.2	0.03±0.03	0±0	0.00
237	gi|67782365	keratin, type II cytoskeletal 7	5.4	51.35	778	11	34.3	1.72±0.23	0.49±0.41	0.29
238	gi|28173564	keratin, type II cytoskeletal 73	6.93	58.89	165	4	8.5	0.22±0.03	0.03±0.03	0.14
239	gi|32567786	keratin, type II cytoskeletal 79	6.75	57.8	229	7	11.6	0.2±0.03	0.03±0.03	0.15
240	gi|6633953	KIAA0097 protein	8.11	225.9	142	6	6.6	no emPAI	0±0	n/a
241	gi|40788922	KIAA0236	8.27	188.7	65	2	5.4	no emPAI	0±0	n/a
242	gi|51105855	KIAA0241 protein	6.22	79.44	78	3	7.9	0±0	no emPAI	n/a
243	gi|3043584	KIAA0530 protein	8.81	173.4	62	2	5.6	no emPAI	0±0	n/a
244	gi|40788287	KIAA0555 protein	5.81	93.85	78	3	9.6	no emPAI	0.05±0.05	n/a

245	gi|40788370	KIAA0801 protein	9.33	120	70	2	9.5	no emPAI	0.04±0.04	n/a
246	gi|7243019	KIAA1319 protein	5.44	137.5	71	2	5.9	no emPAI	0±0	n/a
247	gi|7959231	KIAA1485 protein	6.97	123.5	62	2	8.2	no emPAI	0±0	n/a
248	gi|34328020	KIAA1564 protein	6.47	274.9	89	4	4.1	no emPAI	0±0	n/a
249	gi|219520798	KIAA1671 protein	8.64	196.5	99	3	7.3	no emPAI	0±0	n/a
250	gi|86577784	KIAA1683 protein	10.27	147.3	79	3	8	no emPAI	0±0	n/a
251	gi|24899176	KIAA2005 protein	8.19	184.8	94	3	2.8	no emPAI	0±0	n/a
252	gi|119573383	lamin A/C, isoform CRA_c	8.91	87.32	1591	17	50.9	1.66±0.15	0.21±0.21	0.13
253	gi|27436946	lamin isoform A	6.57	74.1	1464	17	50.5	0±0	0.98±0.80	n/a
254	gi|383792150	lamin isoform D	6.3	63.85	935	11	43.2	0±0	0.82±0.45	n/a
255	gi|5031877	lamin-B1 isoform 1	5.11	66.37	219	6	15.9	0.19±0.03	0.05±0.05	n/a
256	gi|34230	Laminin	5.79	130.8	63	2	7.9	no emPAI	0±0	n/a
257	gi|34234	laminin-binding protein, partial	4.84	31.77	292	9	33	0.28±0.21	0±0.10	0.00
**No.**	**NCBI ID**	**Protein**	**pI**	**MW (kDa)**	**Identification score**	**No. of matched peptides**	**%cov**	**Mock (Mean±SD)**	**Treated (Mean±SD)**	**Ratio (Treated/Mock)**
258	gi|19550955	ligase III, DNA, ATP-dependent	9.15	95.74	79	3	14.3	no emPAI	0±0	n/a
259	gi|4505983	liprin-alpha-1 isoform b	5.91	135.7	104	3	4.8	0±0	0.01±0.01	n/a
260	gi|72534477	low-density lipoprotein receptor-related protein 4	5.14	216.2	73	3	6.8	no emPAI	0.12±0.12	n/a
261	gi|187281	M4 protein	8.99	77.56	114	3	16.9	0.04±0.06	0.40±0.19	9.23
262	gi|2906146	malate dehydrogenase precursor	8.92	35.51	137	4	27.5	0.2±0	0.10±0.05	0.50
263	gi|21735621	malate dehydrogenase, mitochondrial isoform 1 precursor	8.92	35.48	114	3	13.3	0±0	0.09±0.05	n/a
264	gi|139948432	matrix-remodeling-associated protein 5 precursor	8.57	312	77	2	3.8	no emPAI	0.02±0.02	n/a
265	gi|119619130	matrix-remodelling associated 5	8.36	237.5	75	2	5.5	no emPAI	0±0	n/a
266	gi|10945428	membrane-associated guanylate kinase MAGI3	6.04	126	71	2	8.1	no emPAI	0±0	n/a
267	gi|4504143	metabotropic glutamate receptor 5 isoform b precursor	7.59	128.8	61	2	8.7	no emPAI	0±0	n/a
268	gi|3779244	metalloprotease 1	6.56	117.5	89	4	4	no emPAI	0±0	n/a
269	gi|633257783	methionine synthase isoform 3	5.38	96.08	73	3	8.3	no emPAI	2.37±2.37	n/a
270	gi|566559863	methylcytosinedioxygenase TET3	8.04	193.6	69	2	5.8	no emPAI	0±0	n/a
271	gi|189502784	mitochondrial heat shock 60kD protein 1 variant 1	5.83	60.64	1005	17	46	1.42±0.12	0.67±0.63	0.47
272	gi|8131894	mitofilin	5.57	68.15	77	3	13.4	no emPAI	0±0	n/a
273	gi|14625824	moesi0naplastic lymphoma kinase fusion protein	7.61	61.83	99	3	15.9	0±0	0.06±0	n/a
274	gi|4505257	moesin	6.08	67.78	103	3	12	0.05±0	0±0	0.00
275	gi|119616397	monogenic, audiogenic seizure susceptibility 1 homolog (mouse), isoform CRA_f	4.55	681.7	76	2	1.1	no emPAI	0±0	n/a
276	gi|30984034	M-phase phosphoprotein 1	5.75	129.7	66	2	6.2	no emPAI	0±0	n/a
277	gi|292059	MTHSP75	5.97	73.73	188	5	11.5	0.12±0.05	0±0	0.00
278	gi|83367077	mucin-16	5.13	1518	131	3	2	no emPAI	0±0	n/a
**No.**	**NCBI ID**	**Protein**	**pI**	**MW (kDa)**	**Identification score**	**No. of matched peptides**	**%cov**	**Mock (Mean±SD)**	**Treated (Mean±SD)**	**Ratio (Treated/Mock)**
279	gi|1228049	multifunctional protein CAD	6	242.8	85	3	4.9	no emPAI	0±0	n/a
280	gi|28336	mutant beta-actin (beta~-actin)	5.22	41.79	1041	17	57.1	3.31±0.67	2.39±0.99	0.72
281	gi|119600972	MYC binding protein 2, isoform CRA_c	6.63	509.4	64	2	2.6	no emPAI	0±0	n/a
282	gi|62088568	myeloid/lymphoid or mixed-lineage leukemia 2 variant	6.96	293	67	2	2.7	no emPAI	0±0	n/a
283	gi|6731237	myoferlin	5.87	229.6	169	4	12.4	0.05±0.02	0±0	0.00
284	gi|7305053	myoferlin isoform a	5.84	234.6	197	5	11.4	0.04±0.03	0±0	0.00
285	gi|8705240	myosin 5c	7.69	202.7	81	2	3.3	0±0	no emPAI	n/a
286	gi|20338988	myosin heavy chain	6.44	285.1	71	2	5.4	no emPAI	0±0	n/a
287	gi|20338989	myosin heavy chain	6.45	285.1	74	3	5.4	no emPAI	0±0	n/a
288	gi|1235670	myosin VIIA	8.78	254.2	69	2	6.2	no emPAI	0±0	n/a
289	gi|150010558	myosin-15 precursor	5.67	224.5	108	3	5.9	no emPAI	0±0	n/a
290	gi|18650666	myosin-like protein	6.44	285.1	88	4	5.2	no emPAI	0±0	n/a
291	gi|806562	nebulin	9.1	772.7	150	4	5.1	no emPAI	0±0	n/a
292	gi|119631906	nebulin, isoform CRA_c	9.14	348	70	2	3.7	no emPAI	0±0	n/a
293	gi|119631909	nebulin, isoform CRA_f	9.12	777.4	148	4	5.4	no emPAI	0±0	n/a
294	gi|119631910	nebulin, isoform CRA_g	9.1	772.5	71	2	3.8	0±0	no emPAI	n/a
295	gi|22164066	neuroblastoma-amplified protein	5.62	268.3	69	2	2.8	no emPAI	0±0	n/a

296	gi|119580210	neurofilament, heavy polypeptide 200kDa, isoform CRA_c	5.02	64.4	99	3	6.8	0.05±0	0±0	0.00
297	gi|994844	neurofilament-66	5.48	54.88	154	4	13	0.02±0.03	0.04±0.03	2.00
298	gi|35046	NF-M	4.9	102.4	121	4	6.3	0.02±0.02	0±0	0.00
299	gi|119584784	NIMA (never in mitosis gene a)-related kinase 10, isoform CRA_e	7.84	82.83	73	2	5.8	0.04±0	0±0	0.00
300	gi|10048399	non-gastric H+,K+-ATPase	6.12	115.8	79	3	7.1	no emPAI	0±0	n/a
301	gi|641958	non-muscle myosin B	5.44	228.8	68	2	4.8	0±0	2.83±1.42	n/a
302	gi|15213542	NSD1	7.73	284.8	90	4	5.4	0±0	1.87±0.0.94	n/a
**No.**	**NCBI ID**	**Protein**	**pI**	**MW (kDa)**	**Identification score**	**No. of matched peptides**	**%cov**	**Mock (Mean±SD)**	**Treated (Mean±SD)**	**Ratio (Treated/Mock)**
303	gi|1699027	nuclear corepressor KAP-1	5.52	88.48	93	3	5.4	0.04±0.02	0.08±0.08	2.00
304	gi|378404903	nucleolar protein 4-like isoform 1	5.53	74.18	61	2	4.7	no emPAI	0±0	n/a
305	gi|189306	nucleolin	4.59	76.3	128	4	5.9	0.06±0.02	0±0	0.00
306	gi|609342	nucleophosmin-anaplastic lymphoma kinase fusion protein	6.44	75.27	130	3	6.9	0.09±0.08	0.14±0.07	1.50
307	gi|1314308	nucleophosmin-retinoic acid receptor alpha fusion protein NPM-RAR long form	5.85	62.53	148	4	10.7	0.15±0.05	0.09±0.08	0.57
308	gi|68533069	NUMA1 variant protein	5.64	238.7	81	2	2.5	no emPAI	0±0	n/a
309	gi|144226847	obscurin-like protein 1 isoform 1 precursor	5.42	206.8	61	2	3.3	no emPAI	0±0	n/a
310	gi|672717919	ornithine decarboxylase antizyme 3 isoform 3	5.37	23.2	61	2	12.3	no emPAI	0±0	n/a
311	gi|940536	P1 Cdc21 protein	8.39	103	72	2	8.1	0.03±0.01	0±0	0.00
312	gi|2695876	P2Y-like G-protein coupled receptor	9.77	33.38	69	2	3.7	0.1±0.05	0.18±0.18	1.75
313	gi|119601461	pecanex homolog (Drosophila), isoform CRA_d	7.85	181.2	81	2	4.2	no emPAI	0±0	n/a
314	gi|450277	pericentriol material 1	4.99	228.6	90	4	3.3	no emPAI	0±0	n/a
315	gi|189034	perinatal myosin heavy chain	5.64	222.5	70	2	4	0.01±0.01	no emPAI	n/a
316	gi|4505725	peroxisome biogenesis factor 1 isoform 1	5.91	142.8	90	2	5.2	0.02±0	0±0	0.00
317	gi|10518469	phosphoinositide-specific phospholipase C PLC-epsilon	6.08	258.7	106	3	5.3	no emPAI	0±0	n/a
318	gi|68533059	PIK4CA variant protein	6.77	238.5	73	2	4	no emPAI	0±0	n/a
319	gi|1296662	plectin	6.77	238.5	89	4	4.4	0.16±0.06	0.07±0.05	0.45
320	gi|119602580	plectin 1, intermediate filament binding protein 500kDa, isoform CRA_d	5.59	286.1	557	11	11.1	0.06±0.05	0.04±0.04	0.75
321	gi|119599645	plexin D1, isoform CRA_b	6.86	212	62	2	4.9	0±0	no emPAI	n/a
322	gi|2627129	polyubiquitin	7.13	68.45	90	4	7.7	0.1±0	0.01±0	0.05
323	gi|190201	porin	5.87	35.11	119	3	15.6	no emPAI	0±0	n/a
**No.**	**NCBI ID**	**Protein**	**pI**	**MW (kDa)**	**Identification score**	**No. of matched peptides**	**%cov**	**Mock (Mean±SD)**	**Treated (Mean±SD)**	**Ratio (Treated/Mock)**
324	gi|134133226	POTE ankyrin domain family member E	5.83	121.3	606	13	13.9	0.2±0	0±0	0.00
325	gi|145309046	POTE-2 alpha-actin	5.86	121.3	615	14	15	0.2±0	0.07±0.07	0.35
326	gi|10834674	PP3781	9.54	13.59	64	2	29.6	no emPAI	0±0	n/a
327	gi|578823648	PREDICTED: acetyl-CoA carboxylase 2 isoform X7	6	254.9	88	4	4	no emPAI	0±0	n/a
328	gi|578819304	PREDICTED: ankyrin-3 isoform X10	6.03	481.5	78	3	2.8	0±0	0.10±0.09	n/a
329	gi|578820976	PREDICTED: CD44 antigen isoform X13	5.15	44.36	70	2	6.5	0.08±0.08	0.04±0.04	n/a
330	gi|530400856	PREDICTED: centrosomal protein of 290 kDa isoform X4	6.14	234.7	70	2	5	0±0	0.09±0.05	n/a
331	gi|530422334	PREDICTED: dedicator of cytokinesis protein 11 isoform X1	7.79	239	93	3	7	no emPAI	0±0	n/a
332	gi|530383946	PREDICTED: DNA polymerase zeta catalytic subunit isoform X3	8.27	302	104	2	3.9	no emPAI	0±0	n/a
333	gi|530405220	PREDICTED: E3 ubiquitin-protein ligase HERC2 isoform X2	5.88	522.6	118	3	4.2	no emPAI	0±0	n/a
334	gi|530371173	PREDICTED: E3 ubiquitin-protein ligase TRIP12 isoform X5	5.8	213.9	105	3	4.6	no emPAI	0±0	n/a

335	gi|578805102	PREDICTED: fer-1-like protein 5 isoform X1	8.34	239.2	75	2	6.5	no emPAI	0±0	n/a
336	gi|530427501	PREDICTED: heterogeneous nuclear ribonucleoprotein M isoform X1	8.85	75.99	102	3	17.3	0.07±0.07	0±0	0.00
337	gi|530377637	PREDICTED: histone H2A.Z isoform X1	10.65	16.64	104	3	20.4	0.2±0	0±0	0.00
338	gi|530400232	PREDICTED: keratin, type II cuticular Hb3 isoform X1	5.4	49.65	84	3	9.2	0.07±0.04	0±0	0.00
339	gi|530406711	PREDICTED: kinesin-like protein KIF23 isoform X1	8.75	111.6	67	2	6.1	no emPAI	0±0	n/a
340	gi|530383700	PREDICTED: laminin subunit alpha-2 isoform X1	6.13	353.4	83	3	5.2	no emPAI	0±0	n/a
341	gi|530426079	PREDICTED: leucine zipper putative tumor suppressor 3 isoform X1	7.1	74.79	67	2	5.4	no emPAI	0±0	n/a
**No.**	**NCBI ID**	**Protein**	**pI**	**MW (kDa)**	**Identification score**	**No. of matched peptides**	**%cov**	**Mock (Mean±SD)**	**Treated (Mean±SD)**	**Ratio (Treated/Mock)**
342	gi|578823097	PREDICTED: LOW QUALITY PROTEIN: putative uncharacterized protein FLJ45355	11.2	18.24	77	3	26.9	no emPAI	0±0	n/a
343	gi|530365988	PREDICTED: lysosomal-trafficking regulator isoform X2	6.13	423.5	90	4	2.7	no emPAI	0±0	n/a
344	gi|578812715	PREDICTED: metabotropic glutamate receptor 1 isoform X2	9	114.7	76	2	9.2	no emPAI	0±0	n/a
345	gi|530362268	PREDICTED: microtubule-actin cross-linking factor 1 isoform X1	5.22	873	200	4	3.9	no emPAI	0±0	n/a
346	gi|578798816	PREDICTED: microtubule-actin cross-linking factor 1 isoform X18	5.25	855.4	198	5	4.1	no emPAI	0±0	n/a
347	gi|530410154	PREDICTED: myosin phosphatase Rho-interacting protein isoform X1	5.91	247.1	107	3	4.8	0.01±0	0.67±0.62	67.00
348	gi|530420509	PREDICTED: myotubularin-related protein 3 isoform X3	5.89	134.9	65	2	2.7	0±0	no emPAI	n/a
349	gi|578818486	PREDICTED: partitioning defective 3 homolog isoform X16	7.02	130	62	2	2.1	no emPAI	0±0	n/a
350	gi|530378738	PREDICTED: PDZ domain-containing protein 2 isoform X1	7.22	305.3	91	4	3.4	no emPAI	0.01±0.01	n/a
351	gi|530420794	PREDICTED: plexin-B2 isoform X1	6.03	213.8	81	2	8	no emPAI	0±0	n/a
352	gi|578838663	PREDICTED: probable phospholipid-transporting ATPase IG isoform X2	6.27	127.9	81	2	6.5	no emPAI	0.03±0.03	n/a
353	gi|578824299	PREDICTED: protein FAM186B isoform X3	8.98	100.1	92	3	5.7	no emPAI	0±0	n/a
354	gi|578827087	PREDICTED: pyruvate kinase PKM isoform X4	8.18	65.76	79	3	11.7	0±0	0.10±0.05	n/a
355	gi|578821897	PREDICTED: rho guanine nucleotide exchange factor 17 isoform X1	5.7	115.8	75	2	7.9	no emPAI	0±0	n/a
356	gi|578802305	PREDICTED: ryanodine receptor 2 isoform X5	5.7	564.6	64	2	3.2	no emPAI	0±0	n/a
357	gi|578802398	PREDICTED: serine/threonine-protein kinase MRCK alpha isoform X12	5.31	115.2	86	3	5.6	0.03±0	0±0	0.00
**No.**	**NCBI ID**	**Protein**	**pI**	**MW (kDa)**	**Identification score**	**No. of matched peptides**	**%cov**	**Mock (Mean±SD)**	**Treated (Mean±SD)**	**Ratio (Treated/Mock)**
358	gi|530369917	PREDICTED: shugoshin-like 2 isoform X1	7.98	145.3	69	2	8.4	no emPAI	0±0	n/a
359	gi|530387113	PREDICTED: single-stranded DNA-binding protein, mitochondrial isoform X1	10.1	18.55	118	3	20.1	0±0	0.20±0.19	n/a
360	gi|53037374	PREDICTED: succinyl-CoA ligase [GDP-forming] subunit beta, mitochondrial isoform X1	7.57	41.41	76	2	19.3	no emPAI	0±0	n/a
361	gi|578826302	PREDICTED: thyroid receptor-interacting protein 11 isoform X4	5.18	227.4	68	2	5.3	no emPAI	0±0	n/a
362	gi|530406404	PREDICTED: tropomyosin alpha-1 chain isoform X11	4.85	31.73	64	2	14.5	0±0	0.1±0.05	n/a

No.	NCBI ID	Protein	pI	MW (kDa)	Identification score	No. of matched peptides	%cov	Mock (Mean±SD)	Treated (Mean±SD)	Ratio (Treated/Mock)
363	gi|426331707	PREDICTED: tropomyosin alpha-3 chain isoform 6 [Gorilla gorilla gorilla]	4.73	33.2	103	3	17.5	0.10±0.05	0±0	0.00
364	gi|530415144	PREDICTED: ubiquitin-60S ribosomal protein L40 isoform X1	9.18	22.23	142	4	34	0.17±0.25	0.16±0.13	0.92
365	gi|578825530	PREDICTED: uncharacterized protein C14orf37 isoform X1	4.56	72.02	64	2	7.5	no emPAI	0±0	n/a
366	gi|578799996	PREDICTED: uncharacterized protein KIAA1751 isoform X2	6.76	179.5	82	3	3.3	no emPAI	0±0	n/a
367	gi|530370925	PREDICTED: X-ray repair cross-complementing protein 5 isoform X1	8.28	93.46	62	2	6.2	0.04±0.04	no emPAI	n/a
368	gi|578829796	PREDICTED: zinc finger ZZ-type and EF-hand domain-containing protein 1 isoform X4	6.13	211.9	77	3	5	no emPAI	0±0	n/a
369	gi|46198230	P-Rex2	7.5	182.5	101	3	7.5	no emPAI	0±0	n/a
370	gi|359718912	probable E3 ubiquitin-protein ligase HECTD4	5.79	469.7	60	2	2.6	no emPAI	0±0	n/a
371	gi|9507143	probable G-protein coupled receptor 173	9.36	41.45	65	2	10.5	no emPAI	0±0	n/a
372	gi|119609105	prohibitin 2	9.77	32.26	248	7	21.5	0.58±0.07	0.19±0.13	0.32
373	gi|4505773	prohibitin isoform 1	5.57	29.79	635	14	59.2	2.97±0	0.95±0.89	0.32
374	gi|1218058	prohormoneconvertase 5 precursor, partial	7	99.98	89	4	8.1	0.03±0	0±0	0.00
375	gi|24416439	ProSAPiP1 protein	6.36	66.44	68	2	7.7	no emPAI	0±0	n/a
376	gi|12963885	prostate antigen PARIS-1	6.05	104.1	63	2	4	0±0	0.01±0.01	n/a
377	gi|32880208	protein diaphanous homolog 3 isoform b	5.83	98.08	82	3	2.8	0.02±0.02	0±0	0.00
378	gi|506773	protein S100-A9	5.71	13.23	125	4	38.6	0.26±0	0±0	0.00
379	gi|27497737	protein tyrosine phosphatase	6.99	84.42	88	4	12.7	no emPAI	0±0	n/a
380	gi|4505893	proteolipid protein 2	6.79	16.68	61	2	8.6	0.07±0.09	0.10±0.09	1.50
381	gi|4885489	proto-oncogene serine/threonine-protein kinase mos	9.15	37.8	66	2	5.5	no emPAI	0±0	n/a
382	gi|61966919	putative protein FAM47C	6.74	115.3	89	4	6.2	no emPAI	0±0	n/a
383	gi|387011	pyruvate dehydrogenase E1-alpha precursor, partial	8.79	46.24	70	2	8	no emPAI	0±0	n/a
384	gi|119620329	RAB1A, member RAS oncogene family, isoform CRA_f	8.81	28.13	79	3	23	0.12±0	0±0	0.00
385	gi|119620331	RAB1A, member RAS oncogene family, isoform CRA_h	8.71	11.74	68	2	29.4	0.29±0	0±0	0.00
386	gi|355390217	Ras-related protein Rab-15 isoform AN2	6.27	13.77	68	3	18.5	0.25±0	0±0	0.00
387	gi|13569962	ras-related protein Rab-1B	5.55	22.16	82	2	22.9	0.15±0	0±0	0.00
388	gi|5803135	ras-related protein Rab-35 isoform 1	8.52	23.01	72	2	19.9	0.15±0	0±0	0.00
389	gi|47116772	RecName: Full=Latrophilin-3; AltName: Full=Calcium-independent alpha-latrotoxin receptor 3; Short=CIRL-3; AltName: Full=Lectomedin-3; Flags: Precursor	5.99	161.7	60	2	2.8	no emPAI	0±0	n/a
390	gi|338817989	RecName: Full=Microtubule-actin cross-linking factor 1, isoforms 1/2/3/5; AltName: Full=620 kDa actin-binding protein; Short=ABP620; AltName: Full=Actin cross-linking family protein 7; AltName: Full=Macrophin-1; AltName: Full=Trabeculin-alpha [Homo	5.28	837.8	111	2	3.5	0±0	0.21±0.11	n/a
No.	NCBI ID	Protein	pI	MW (kDa)	Identification score	No. of matched peptides	%cov	Mock (Mean±SD)	Treated (Mean±SD)	Ratio (Treated/Mock)
391	gi|74739412	RecName: Full=Putative beta-actin-like protein 3; AltName: Full=Kappa-	5.28	837.8	128	3	3.2	0.35±0	0.08±0.08	0.23

		actin; AltName: Full=POTE ankyrin domain family member K								
392	gi|158518381	RecName: Full=Putative heat shock 70 kDa protein 7; AltName: Full=Heat shock 70 kDa protein B	7.72	40.22	68	2	6	0.08±0	0±0	0.00
393	gi|136066	RecName: Full=Triosephosphateisomerase; Short=TIM; AltName: Full=Triose-phosphate isomerase [Oryctolaguscuniculus]	7.1	26.61	124	4	28.6	0.27±0	0.07±0.06	0.24
394	gi|90101849	RecName: Full=Uromodulin-like 1; AltName: Full=Olfactorin; Flags: Precursor	5.75	144.2	85	3	5.8	no emPAI	0±0	n/a
395	gi|21361458	rho guanine nucleotide exchange factor 17	5.9	221.5	69	2	4.9	no emPAI	0±0	n/a
396	gi|808090	ribosomal protein L21	10.26	18.5	71	2	9.4	0±0	0.09±0.09	n/a
397	gi|119584991	ribosomal protein SA, isoform CRA_c	8.37	19.63	227	6	53.6	0.44±0.44	0.61±0.31	1.39
398	gi|307383	RNA helicase A	6.17	142	93	3	10.7	no emPAI	0.03±0.02	n/a
399	gi|337722	ryanodine receptor	5.19	564.1	78	3	3.3	0±0	0.06±0.03	n/a
400	gi|337930	scar protein	9.95	27.39	62	2	10.2	0.04±0.06	0±0	0.00
401	gi|33313303	SCCA1/SCCA2 fusion protein	6.19	44.74	159	4	21.3	0.15±0	0±0	0.00
402	gi|33317676	SCCA2/SCCA1 fusion protein isoform 1	5.99	44.62	127	4	19.7	0.07±0	0±0	0.00
403	gi|21307630	septin 1	5.56	42.39	64	2	4	no emPAI	0±0	n/a
404	gi|119605885	serine/arginine repetitive matrix 2, isoform CRA_c	12.09	288.2	175	5	6.9	no emPAI	0±0	n/a
405	gi|5902076	serine/arginine-rich splicing factor 1 isoform 1	10.37	27.73	162	4	27.4	0.47±0	0.02±0.01	0.04

No.	NCBI ID	Protein	pI	MW (kDa)	Identification score	No. of matched peptides	%cov	Mock (Mean±SD)	Treated (Mean±SD)	Ratio (Treated/Mock)
406	gi|28076869	serpin B4 isoform 1	5.86	44.83	119	3	20.3	0.07±0	0±0	0.00
407	gi|19263767	Similar to cytoskeleton-associated protein 4, partial	5.51	62.02	183	5	15	0.13±0.03	0.23±0.11	1.77
408	gi|4506975	solute carrier family 12 member 2 isoform 1	5.98	131.4	66	2	8.4	no emPAI	0±0	n/a
409	gi|119610275	solute carrier family 25 (mitochondrial carrier; adenine nucleotide translocator), member 5, isoform CRA_b	9.6	27.61	350	9	42.2	1.06±0.11	0.75±0.70	0.70
410	gi|15928608	Solute carrier family 25 (mitochondrial carrier; adenine nucleotide translocator), member 6	9.76	32.91	343	9	31.9	0.52±0.37	0.58±0.55	1.12
411	gi|119568122	spectrin repeat containing, nuclear envelope 1, isoform CRA_k	5.38	1008	135	5	3.7	no emPAI	0±0	n/a
412	gi|6006515	spliceosomal protein SAP 130	5.15	135.5	87	4	3.5	0±0	0.02±0.01	n/a
413	gi|119627828	splicing factor proline/glutamine-rich (polypyrimidine tract binding protein associated), isoform CRA_c	9.41	67.77	100	2	11.7	0.03±0.02	0.10±0.05	4.00
414	gi|4826998	splicing factor, proline- and glutamine-rich	9.45	76.1	139	4	10.7	0.04±0.04	0.07±0.06	1.50
415	gi|29825635	squamous cell carcinoma antigen 2	5.99	44.76	98	3	28.5	no emPAI	0±0	n/a
416	gi|260436922	suprabasin isoform 1 precursor	6.5	60.51	64	2	5.6	0.05±0	0±0	0.00
417	gi|27451602	tau-tubulin kinase	6.58	182.4	107	3	7.2	0.02±0.01	0±0	0.00
418	gi|124376546	T-box 18	9.06	64.81	70	2	6.4	no emPAI	0±0	n/a
419	gi|1848277	telomerase-associated protein TP-1	8.17	290.2	92	4	3.4	no emPAI	0±0	n/a
420	gi|188528648	tenascin-X isoform 1 precursor	5.05	457.9	116	3	4.9	no emPAI	0±0	n/a
421	gi|58331272	terminal uridylyltransferase 7 isoform 1	6.4	171.1	66	2	3.4	no emPAI	0±0	n/a
422	gi|4838145	tetrodotoxin-resistant voltage-gated sodium channel	5.67	220.4	63	2	2.1	no emPAI	0±0	n/a
423	gi|4378531	thioredoxinreductase	7.24	56.49	76	2	13.9	no emPAI	0±0	n/a
424	gi|5802974	thioredoxin-dependent peroxide	7.67	27.68	94	3	16	0.26±0	0±0	0.00
No.	NCBI ID	Protein	pI	MW (kDa)	Identification score	No. of matched peptides	%cov	Mock (Mean±SD)	Treated (Mean±SD)	Ratio (Treated/Mock)

425	gi|27764565	reductase, mitochondrial isoform a precursor	7.55	59.33	110	3	5.9	0.07±0.05	0.03±0.03	0.41
		TPA_exp: keratin 5c								
426	gi|257467636	TPR and ankyrin repeat-containing protein 1	6.34	336	81	2	3	no emPAI	0±0	n/a
427	gi|31652242	transcription factor 20 isoform 2	9.19	209.5	86	3	5.3	no emPAI	0±0	n/a
428	gi|460789	transformation upregulated nuclear protein	5.13	51.04	92	4	12.5	0.06±0	0±0	0.00
429	gi|119622920	transient receptor potential cation channel, subfamily M, member 5, isoform CRA_b, partial	6.27	132.4	65	2	1.8	no emPAI	0.07±0.07	n/a
430	gi|6005942	transitional endoplasmic reticulum ATPase	5.14	89.27	75	2	2.7	0±0	0.11±0.07	n/a
431	gi|37267	transketolase	7.9	67.75	73	3	6.6	0.1±0	0.03±0.02	0.25
432	gi|1903414	translation repressor NAT1	6.57	102.1	63	2	7.5	no emPAI	0±0	n/a
433	gi|118421085	treslin	9	210.7	71	2	9.6	no emPAI	0±0	n/a
434	gi|12311759	type I hair keratin 8	4.79	50.46	77	2	6.1	0±0	0.04±0.04	n/a
435	gi|31074641	type I inner root sheath specific keratin 25 irs3	5.06	49.79	159	4	12	0.21±0	0±0	0.00
436	gi|31074643	type I inner root sheath specific keratin 25 irs4	5.33	50.56	171	4	13.1	0.21±0	0.04±0.03	0.17
437	gi|2950168	type I keratin	4.87	47.2	71	2	3.8	0.07±0	0.04±0.04	0.50
438	gi|1195531	type I keratin 16	4.99	51.21	457	13	29.4	0.55±0	0±0	0.00
439	gi|22798968	type II hair keratin 2	6.4	56.65	63	2	1.4	0.03±0.03	0±0	0.00
440	gi|4885409	vigilin isoform a	6.43	141.4	73	3	5.1	no emPAI	0±0	n/a
441	gi|47115317	VIM	5.09	53.55	1677	18	74.5	4.37±3.34	5.76±2.37	1.32
442	gi|5030431	vimentin	4.82	41.54	1292	17	62.1	5.53±1.04	0±0	0.00
443	gi|62414289	vimentin	5.06	53.62	1880	18	74	4.62±4.62	3.08±3.08	0.67
444	gi|25188179	voltage-dependent anion-selective channel protein 3 isoform 1	6.6	110.2	77	3	6.2	0±0	0.11±0.05	n/a
**No.**	**NCBI ID**	**Protein**	**pI**	**MW (kDa)**	**Identification score**	**No. of matched peptides**	**%cov**	**Mock (Mean±SD)**	**Treated (Mean±SD)**	**Ratio (Treated/Mock)**
445	gi|14970562	WDR9 protein, form A	8.73	262.8	75	2	3.5	no emPAI	0±0	n/a
446	gi|1160186	white	8.04	75.12	68	2	8.8	0±0	no emPAI	n/a
447	gi|119372317	xin actin-binding repeat-containing protein 2 isoform 1	5.95	401.9	68	2	2.6	no emPAI	0±0	n/a
448	gi|4503841	X-ray repair cross-complementing protein 6 isoform 1	6.23	69.8	89	4	13	0±0	0.03±0.02	n/a
449	gi|1205999	YRRM, partial	11.73	4.474	66	2	69.8	no emPAI	0±0	n/a
450	gi|18652806	zinc-induced metallothionein 2A/1A hybrid	8.23	6.113	62	2	78.7	no emPAI	0±0	n/a

NCBI = National Center for Biotechnology Information. %Cov. = %Sequence covage [(number of the mathched residues/total number of residues in the entire sequence) x 100%]. DIV/0 = Divide by zero. emPAI = Exponentially modified protein abundance index. n/a = not available.

Supplement 4 . Alteration of structural molecule activity proteins in EA.hy926 cells during DENV2 infection and TNF-α treatment.

No.	NCBI ID	Protein	pI	MW (kDa)	Identification score	No. of matched peptides	%cov	Mock (Mean±SD)	Treated (Mean±SD)	Ratio (Treated/Mock)
1	gi|20149844	Chain A, Human Vimentin Coil 1a Fragment (1a)	4.6	4.646	188	7	76.9	9.3±0	no emPAI	n/a
2	gi|374414590	Chain A, Crystal Structure Of Stabilised Vimentin Coil2 Fragment	5.41	8.667	307	9	79.2	2.52±0.53	no emPAI	n/a
3	gi|4501881	actin, alpha skeletal muscle	5.23	42.024	705	15	41.4	1.84±0.38	no emPAI	n/a

4	gi|181573	cytokeratin 8	5.52	53.529	877	11	30.7	1.18±0.21	no emPAI	n/a
5	gi|635576697	Chain B, Crystal Structure Of Schizosaccharomyces Pombe Sst2 Catalytic Domain And Ubiquitin	6.78	8.971	94	3	63	0.31±0.44	no emPAI	n/a
6	gi|31074631	keratin 1b	5.73	61.763	234	7	9.9	0.23±0	no emPAI	n/a
7	gi|145580001	Chain A, Crystal Structure Of A Fragment Of The Plakin Domain Of Plectin	7.18	42.13	250	7	24.3	0.12±0	no emPAI	n/a
8	gi|219524	cardiac alpha-myosin heavy chain	5.63	223.414	114	3	4.5	0.01±0	no emPAI	n/a
9	gi|189034	perinatal myosin heavy chain	5.64	222.525	70	2	4	0.01±0.01	no emPAI	n/a
10	gi|212293358	actinin alpha 1 isoform 3	5.38	107.074	157	4	5.1	0±0	0.03±0.02	n/a
11	gi|12053672	beta-myosin heavy chain	5.63	222.963	72	2	6.1	0±0	0.11±0.06	n/a
12	gi|29727	cardiac beta myosin heavy chain	5.62	222.849	79	3	5.9	0±0	no emPAI	n/a
13	gi|181390	cytokeratin 2	8.38	65.83	89	4	5	0±0	0.03±0.02	n/a
14	gi|386848	keratin	5.09	51.63	86	3	4.9	0±0	0.03±0.03	n/a
15	gi|186685	keratin type 16	4.97	50.668	78	3	6.4	0±0	0.04±0.04	n/a
16	gi|27436946	lamin isoform A	6.57	74.095	1464	17	50.5	0±0	0.98±0.80	n/a
17	gi|383792150	lamin isoform D	6.3	63.854	935	11	43.2	0±0	0.82±0.45	n/a
18	gi|8705240	myosin 5c	7.69	202.666	81	2	3.3	0±0	no emPAI	n/a
19	gi|641958	non-muscle myosin B	5.44	228.798	68	2	4.8	0±0	2.83±1.42	n/a
		RecName: Full=Microtubule-actin cross-linking factor 1, isoforms 1/2/3/5; AltName: Full=620 kDa;								

No.	NCBI ID	Protein	pI	MW (kDa)	Identification score	No. of matched peptides	%cov	Mock (Mean±SD)	Treated (Mean±SD)	Ratio (Treated/Mock)
20	gi|338817989	actin-binding protein; Short=ABP620AltName: Full=Actin cross-linking family protein 7; AltName: Full=Macrophin-1; AltName: Full=Trabeculin-alpha [Homo	5.28	837.787	111	2	3.5	0±0	0.21±0.11	n/a
21	gi|808090	ribosomal protein L21	10.26	18.503	71	2	9.4	0±0	0.09±0.09	
22	gi|12311759	type I hair keratin 8	4.79	50.457	77	2	6.1	0±0	0.04±0.04	n/a
23	gi|51476571	hypothetical protein	5.24	122.013	61	2	8.4	no emPAI	1.34±1.34	n/a
24	gi|4502247	armadillo repeat protein deleted in velo-cardio-facial syndrome	6.38	104.577	99	3	8.5	no emPAI	0±0	n/a
25	gi|38348729	coiled-coil domain-containing protein 171	6.37	152.714	98	3	7.9	no emPAI	0±0	n/a
26	gi|222144249	dynein heavy chain domain-containing protein 1 isoform 1	6.25	533.305	104	3	2.6	no emPAI	0±0	n/a
27	gi|46559358	fibrillin 1	4.81	312.006	101	3	9.5	no emPAI	0±0	n/a
28	gi|260268505	FLJ00343 protein	5.77	281.288	145	4	7.3	no emPAI	0±0	n/a
29	gi|6633953	KIAA0097 protein	8.11	225.893	142	6	6.6	no emPAI	0±0	n/a
30	gi|7243019	KIAA1319 protein	5.44	137.518	71	2	5.9	no emPAI	0±0	n/a
31	gi|20338988	myosin heavy chain	6.44	285.056	71	2	5.4	no emPAI	0±0	n/a
32	gi|20338989	myosin heavy chain	6.45	285.06	74	3	5.4	no emPAI	0±0	n/a
33	gi|1235670	myosin VIIA	8.78	254.245	69	2	6.2	no emPAI	0±0	n/a
34	gi|150010558	myosin-15 precursor	5.67	224.479	108	3	5.9	no emPAI	0±0	n/a
35	gi|18650666	myosin-like protein	6.44	285.075	88	4	5.2	no emPAI	0±0	n/a
36	gi|144226847	obscurin-like protein 1 isoform 1 precursor	5.42	206.817	61	2	3.3	no emPAI	0±0	n/a
37	gi|61966919	putative protein FAM47C	6.74	115.266	89	4	6.2	no emPAI	0±0	n/a
38	gi|21307630	septin 1	5.56	42.389	64	2	4	no emPAI	0±0	n/a
39	gi|119372317	xin actin-binding repeat-containing protein 2 isoform 1	5.95	401.866	68	2	2.6	no emPAI	0±0	n/a
40	gi|181402	epidermal cytokeratin 2	8.07	65.825	336	9	9.1	0.41±0	4.75±4.41	11.51

No.	NCBI ID	Protein	pI	MW (kDa)	Identification score	No. of matched peptides	%cov	Mock (Mean±SD)	Treated (Mean±SD)	Ratio (Treated/Mock)
41	gi|4504927	keratin, type I cuticular Ha6	4.9	52.214	91	4	6.2	0.03±0.03	0.06±0.03	2.00
42	gi|994844	neurofilament-66	5.48	54.875	154	4	13	0.02±0.03	0.04±0.03	2.00
43	gi|47115317	VIM	5.09	53.547	1677	18	74.5	4.37±3.34	5.76±2.37	1.32
44	gi|229597861	Chain A, X-Ray Crystal Structure Of Coil 1a Of Human Vimentin	4.35	4.674	198	5	76.9	4.1±3.72	5.24±2.85	1.28
45	gi|15928608	Solute carrier family 25 (mitochondrial carrier; adenine	9.76	32.905	343	9	31.9	0.52±0.37	0.58±0.55	1.12

No.	NCBI ID	Protein	pI	MW (kDa)	Identification score	No. of matched peptides	%cov	Mock (Mean±SD)	Treated (Mean±SD)	Ratio (Treated/Mock)
		nucleotide translocator), member 6								
46	gi|530537677	Chain E, Structure Of An Rsp5xubxsna3 Complex: Mechanism Of Ubiquitin Ligation And Lysine Prioritization By A Hect E3	7.06	9.559	127	4	73.5	0.51±0.73	0.43±0.35	0.84
47	gi|339920	ADP/ADT translocator protein	9.84	33.127	257	8	30	0.47±0	0.39±0.37	0.83
48	gi|28336	mutant beta-actin (beta~-actin)	5.22	41.786	1041	17	57.1	3.31±0.67	2.39±0.99	0.72
49	gi|62414289	vimentin	5.06	53.619	1880	18	74	4.62±4.62	3.08±3.08	0.67
50	gi|4501887	actin, cytoplasmic 2	5.31	41.766	1170	17	61.6	4.02±0.78	2.65±1.10	0.66
51	gi|2950168	type I keratin	4.87	47.202	71	2	3.8	0.07±0	0.04±0.04	0.50
52	gi|30311	cytokeratin 18 (424 AA)	5.27	47.305	1072	17	56.6	1.9±0.40	0.88±0.73	0.46
53	gi|1296662	plectin	6.77	238.544	89	4	4.4	0.16±0.06	0.07±0.05	0.45
54	gi|159162145	Chain A, Rotamer Strain As A Determinant Of Protein Structural Specificity	6.56	8.56	95	3	61.8	0.6±0.27	0.26±0.17	0.42
55	gi|11935049	keratin 1	8.16	66.027	353	9	23.6	0.26±0.37	0.11±0.09	0.40
56	gi|179247	ADP/ATP carrier protein	9.71	32.96	308	9	36.6	0.67±0	0.27±0.25	0.39
57	gi|67782365	keratin, type II cytoskeletal 7	5.4	51.354	778	11	34.3	1.72±0.23	0.49±0.41	0.29
58	gi|5031877	lamin-B1 isoform 1	5.11	66.368	219	6	15.9	0.19±0.03	0.05±0.05	0.27
59	gi|74739412	RecName: Full=Putative beta-actin-like protein 3; AltName: Full=Kappa-actin; AltName: Full=POTE ankyrin domain family member K	5.28	837.787	128	3	3.2	0.35±0	0.08±0.08	0.23
60	gi|31074643	type I inner root sheath specific keratin 25 irs4	5.33	50.564	171	4	13.1	0.21±0	0.04±0.03	0.17
61	gi|32567786	keratin, type II cytoskeletal 79	6.75	57.8	229	7	11.6	0.2±0.03	0.03±0.03	0.15
62	gi|28173564	keratin, type II cytoskeletal 73	6.93	58.887	165	4	8.5	0.22±0.03	0.03±0.03	0.14
63	gi|34035	cytokeratin 18 (232 AA)	4.92	26.434	302	9	39.2	0.71±0.1	0.10±0.10	0.13
64	gi|4557701	keratin, type I cytoskeletal 17	4.97	48.076	208	4	16.7	0.22±0	0.03±0.02	0.11
65	gi|2627129	polyubiquitin	7.13	68.448	90	4	7.7	0.1±0	0.01±0	0.05
66	gi|12803709	Keratin 14	5.09	51.619	402	11	23.5	0.45±0	0.01±0.02	0.03
67	gi|908803	keratin type II	8.09	60.186	476	14	27	0.64±0.04	0.02±0.03	0.03
68	gi|325533397	Chain A, Human Lamin A Coil 2b Fragment	5.46	8.661	231	7	60.8	1.69±1.02	0.03±0.03	0.02
69	gi|4506631	60S ribosomal protein L30	9.65	12.776	95	2	36.5	0.44±0.17	0±0	0.00
70	gi|31615803	Chain A, Synthetic Ubiquitin With Fluoro-Leu At 50 And 67	6.56	8.555	128	3	77.6	1.26±0.38	0±0.47	0.00
71	gi|5031635	cofilin-1	8.22	18.491	105	3	25.9	0.27±0.19	0±0.08	0.00
72	gi|435476	cytokeratin 9	5.19	62.092	437	11	25.4	0.59±0	0±0	0.00
73	gi|30722305	hypothetical protein	5.66	222.834	168	4	9.6	0.01±0	0±0	0.00
74	gi|21961605	Keratin 10	5.09	58.792	487	14	33.6	0.69±0.09	0±0	0.00
75	gi|18999435	Keratin 5	7.59	62.34	393	9	19.2	0.38±0.03	0±0	0.00
76	gi|4504935	keratin, type II cuticular Hb5 isoform 1	6.28	55.766	107	3	13.2	0.03±0.03	0±0	0.00
77	gi|34234	laminin-binding protein, partial	4.84	31.774	292	9	33	0.28±0.21	0±0.10	0.00
78	gi|4505257	moesin	6.08	67.778	103	3	12	0.05±0	0±0	0.00
79	gi|35046	NF-M	4.9	102.386	121	4	6.3	0.02±0.02	0±0	0.00
80	gi|32880208	protein diaphanous homolog 3 isoform b	5.83	98.078	82	3	2.8	0.02±0.02	0±0	0.00
81	gi|337930	scar protein	9.95	27.386	62	2	10.2	0.04±0.06	0±0	0.00
82	gi|31074641	type I inner root sheath specific keratin 25 irs3	5.06	49.793	159	4	12	0.21±0	0±0	0.00
83	gi|1195531	type I keratin 16	4.99	51.206	457	13	29.4	0.55±0	0±0	0.00
84	gi|22798968	type II hair keratin 2	6.4	56.646	63	2	1.4	0.03±0.03	0±0	0.00
85	gi|5030431	vimentin	4.82	41.537	1292	17	62.1	5.53±1.04	0±0	0.00

NCBI = National Center for Biotechnology Information. %Cov. = %Sequence covage [(number of the mathched residues/total number of residues in the entire sequence) x 100%]. DIV/0 = Divide by zero. emPAI = Exponentially modified protein abundance index. n/a = not available.
